# Supplementary material for: Safety and pharmacokinetics-pharmacodynamics of a shorter tuberculosis treatment with high-dose pyrazinamide and rifampicin: a study protocol of a phase II clinical trial (HighShort-RP)
Source: BMJ Open. 2022 Mar 9;12(3):e054788. doi: 10.1136/bmjopen-2021-054788 (PMC8915351; doi:10.1136/bmjopen-2021-054788)
Supplement: Author's manuscript [file bmjopen-2021-054788.draft_revisions.pdf]

BMJ Open

BMJ Open is committed to open peer review. As part of this commitment we make the peer review history of every article we publish publicly available.

When an article is published we post the peer reviewers' comments and the authors' responses online. We also post the versions of the paper that were used during peer review. These are the versions that the peer review comments apply to.

The versions of the paper that follow are the versions that were submitted during the peer review process. They are not the versions of record or the final published versions. They should not be cited or distributed as the published version of this manuscript.

BMJ Open is an open access journal and the full, final, typeset and author-corrected version of record of the manuscript is available on our site with no access controls, subscription charges or pay-per-view fees (http://bmjopen.bmj.com).

If you have any questions on BMJ Open's open peer review process please email info.bmjopen@bmj.com

BMJ Open

Safety and pharmacokinetics-pharmacodynamics of a shorter tuberculosis treatment with high-dose pyrazinamide and rifampicin – a study protocol of a phase II clinical trial (HighShort-RP)

Journal:	*BMJ Open*
Manuscript ID	bmjopen-2021-054788
Article Type:	Protocol
Date Submitted by the Author:	22-Jun-2021

Complete List of Authors:	Ekqvist, David; Linköping University Hospital, Department of Infectious Diseases<br>Bornefall, Anna; Linköping University Hospital, Department of Infectious Diseases<br>Nordqvist, Daniel; Linköping University Hospital, Department of Infectious Diseases<br>Sönnerbrandt, Martina; Linköping University Hospital, Department of Infectious Diseases<br>Nordvall, Michaela Jonsson; Linköping University, Department of Clinical Microbiology, and Department of Biomedical and Clinical Sciences<br>Fredrikson, Mats; Linköping University, Forum Östergötland<br>Carlsson, Björn; Linköping University, Department of Clinical Pharmacology, and Department of Biomedical and Clinical Sciences<br>Sandstedt, Mårten; Linköping University, Department of Radiology in Linköping, and Department of Health, Medicine and Caring Sciences<br>Simonsson, Ulrika; Uppsala University, Department of Pharmaceutical Biosciences<br>Alffenaar, Jan-Willem; University of Syndney, Schoold of Pharmacy, Faculty of Medicine and Health; University of Sydney SDN, Bashir Institute of Infectious Diseases and Biosecurity<br>Paues, Jakob; Linkopings universitet, Department of Infectious Diseases, and Department of Biomedical and Clinical Sciences<br>Niward, Katarina; Linkopings universitet, Department of Infectious Diseases, and Department of Biomedical and Clinical Sciences

Keywords:	Tuberculosis < INFECTIOUS DISEASES, Clinical trials < THERAPEUTICS, Adverse events < THERAPEUTICS, CLINICAL PHARMACOLOGY

SCHOLARONE™ Manuscripts

Safety and pharmacokinetics-pharmacodynamics of a shorter tuberculosis treatment with high-dose pyrazinamide and rifampicin – a study protocol of a phase II clinical trial (HighShort-RP)

David Ekqvist[1], Anna Bornefall[1], Daniel Nordqvist[1], Martina Sönnerbrandt[1], Michaela Jonsson Nordvall[2], Mats Fredrikson[3], Björn Carlsson[4], Mårten Sandstedt[5], Ulrika S. H. Simonsson[6], Jan-Willem Alffenaar[7,8,9], Jakob Paues[10], Katarina Niward*[10]

1. Department of Infectious Diseases, Linköping University Hospital, Linköping, Sweden
2. Department of Clinical Microbiology, and Department of Biomedical and Clinical Sciences, Linköping University, Linköping, Sweden
3. Forum Östergötland, Linköping University, Linköping, Sweden
4. Department of Clinical Pharmacology, and Department of Biomedical and Clinical Sciences, Linköping University, Linköping, Sweden
5. Department of Radiology in Linköping, and Department of Health, Medicine and Caring Sciences, Linköping University, Linköping, Sweden
6. Department of Pharmaceutical Biosciences, Uppsala University, Sweden
7. University of Sydney, Faculty of Medicine and Health, School of Pharmacy, Sydney, Australia
8. Westmead hospital, Sydney, Australia
9. Marie Bashir Institute of Infectious Diseases and Biosecurity, University of Sydney, Sydney, Australia
10. Department of Infectious Diseases, and Department of Biomedical and Clinical Sciences, Linköping University, Linköping, Sweden

*Corresponding author: Katarina Niward (katarina.niward@liu.se)

Key words: pulmonary tuberculosis, pyrazinamide, rifampicin, pharmacokinetic-pharmacodynamics (PK-PD), adverse event (AE), time to culture positivity (TTP)

Word count: 3996

For peer review only

ABSTRACT

Introduction So far, attempts to shorten treatment of drug-susceptible TB have failed. Dose optimisation of rifampicin and pyrazinamide seems a viable strategy to shorten treatment and prevent relapse, but safety and efficacy remains to be confirmed. This clinical trial aims to explore safety and pharmacokinetics-pharmacodynamics of a high-dose pyrazinamide-rifampicin regimen to shorten TB treatment.

Methods and analysis Adult patients with pulmonary TB admitted to six hospitals in Sweden and subjected to receive first-line treatment, are included. Patients are randomised (1:3) to either 6-month standardised TB treatment or a 4-month regimen based on high-dose pyrazinamide (40 mg/kg) and rifampicin (35 mg/kg) along with standard doses of isoniazid and ethambutol. Plasma samples for measurement of drug exposure determined by liquid chromatography tandem-mass spectrometry are obtained at 0, 1, 2, 4, 6, 8, 12 and 24 h, at day 1 and 14. Maximal drug concentration (C_{max}) and area under the concentration-time curve ($AUC_{0\text{-}24h}$) are estimated by non-compartmental analysis. Conditions for early model-informed precision dosing of high-dose pyrazinamide and rifampicin are pharmacometrically explored. Adverse drug effects are monitored throughout the study and graded according to Common Terminology Criteria for Adverse Events v 5.0. Early bactericidal activity is assessed by time to positivity in BACTEC MGIT 960 of induced sputum collected at day 0, 5, 8, 15 and week 8. Minimum inhibitory concentrations of first-line drugs are determined using broth microdilution. Disease severity is assessed with X-ray grading and a validated clinical scoring tool (TBscore II). Clinical outcome is registered according to WHO definitions in addition to occurrence of relapse after end of treatment.

For peer review only

Ethics and dissemination The study is approved by the Swedish Ethical Review Authority and the Swedish Medical Products Agency. Informed written consent is collected before participation. The study results will be submitted to a peer-reviewed journal.

Trial registration number NCT04694586; Pre-results.

ARTICLE SUMMARY

Strengths and limitations of this study

- This phase II clinical trial of patients with drug-susceptible pulmonary tuberculosis (TB) will provide data on safety and early bactericidal activity in relation to drug exposure of an experimental shorter TB regimen based on high-dose pyrazinamide and rifampicin.
- The patients' drug exposure will be related to individual *Mycobacterium tuberculosis* minimal inhibitory concentrations (MICs), exploring pharmacokinetics-pharmacodynamics indices during high-dose pyrazinamide and rifampicin.
- Opportunities for early model-informed precision dosing after the first dose will be explored by pharmacokinetic modelling.
- A limitation of the study is the small sample size, due to an extensive and resource demanding study protocol, although this is in accordance with phase II studies and will partly be compensated for by using rich data.

For peer review only

INTRODUCTION

Current treatment of drug-susceptible TB is based on recommendations by the World Health Organization (WHO) and originates from the 1970s (1, 2). During the first two months (intensive phase) daily doses of isoniazid 5 mg/kg , rifampicin 10 mg/kg, pyrazinamide 25 mg/kg, and ethambutol 15 mg/kg are given, followed by a continuation phase of isoniazid and rifampicin (1). This 6-month multi-drug regimen is intended to prevent acquired drug resistance, therapy failure and relapse (3). Although effective, the long treatment duration increases the risk of non-compliance. Hence shorter and more effective regimens with preserved safety profile are needed as part of the global strategy to eliminate TB. Several attempts to shorten TB treatment below 6 months have been unsuccessful due to increased frequency of relapse (4-7). Only, a pooled analysis of patient-level data from 4-month fluoroquinolone-containing regimens identified non-inferiority compared with standard treatment in participants with minimal non-cavitary disease (8).

Accumulating data from clinical trials exploring higher doses of rifampicin (9-12) have shown increased bactericidal activity with potential to shorten TB treatment corroborated by results from pharmacokinetic-pharmacodynamic (PK-PD) modelling and simulation (13, 14). Lately, pyrazinamide has attained increasing attention due to its potent sterilising activity with the ability to eliminate intracellular resting and slowly replicating *Mycobacterium tuberculosis* (*Mtb*) so-called “persisters” (15). Once the drug was added to standard TB treatment in the early 1980s the treatment length could be shortened from 9 to 6 months without increase in relapse (2, 16). Efforts to further shorten TB treatment from 6 to 4 months by simply prolonging the duration of pyrazinamide was unsuccessful because of increased frequency of relapse (17). Observational studies of more than 100 patients with pulmonary TB (18, 19) and a recent meta-analysis (20) have shown that unsuccessful treatment outcome was associated with suboptimal pyrazinamide exposure. Pharmacokinetic studies in patients

For peer review only

with pulmonary TB have shown lower than recommended drug concentration levels (21) with standard dosing of both rifampicin (42-80%) and pyrazinamide (27-53%) (22, 23). In a large PK-PD study evaluating patient-level data from three phase II clinical trials with pulmonary TB patients where the majority had cavitary disease, participants received rifampicin (range 10-35 mg/kg), pyrazinamide (range 20-30 mg/kg) and two companion drugs (24). The results showed that pyrazinamide's induction of culture negativity increased with augmented drug exposure. Hepatotoxicity was rare and not associated with pyrazinamide exposure. Furthermore, pharmacokinetic modelling suggested that a massive dose of pyrazinamide (>4500 mg) or increasing both pyrazinamide and rifampicin doses would be required to reach microbiological targets associated with treatment-shortening such as 90-95% culture conversion rate by 2 months of treatment (24). Rifampicin doses of at least three times the standard dose combined with isoniazid, pyrazinamide and ethambutol was safely administered to 63 patients with pulmonary TB for 12 weeks (10). Rifampicin 40 mg/kg was recently identified as the highest tolerated dose with dose limiting factors reported as gastrointestinal disorders, pruritis, hyperbilirubinemia and jaundice (25). However, the safety of simultaneously administered high-dose pyrazinamide and rifampicin remains to be explored. Our study aims to investigate PK-PD data, safety and tolerability of a 4-month first-line treatment with combined high dosing of pyrazinamide and rifampicin to a selected group of patients with non-advanced pulmonary TB.

METHODS AND ANALYSIS

Study design

We are conducting a multicentre open-label randomised controlled phase II study. The study protocol conforms with SPIRIT (Standard Protocol Items: Recommendations for Interventional Trials), an international format for interventional trials (26, 27). The primary

For peer review only

outcome is $AUC_{0\text{-}24h}$ of pyrazinamide in the intervention arm and the main secondary outcome is safety.

Study setting

The study is carried out at infectious disease departments in six hospitals in Sweden, three tertiary level hospitals and three secondary level hospitals. The number of hospital beds in the hospitals range from 310 to 1340. The incidence of TB in Sweden is 4.75/100 000 (2019) and the vast majority (85%) of patients are migrants that have acquired their infection prior to arriving to Sweden (28). The number of new culture positive pulmonary TB cases during 2019 was 93 in total within the regions served by the six hospitals included in the study (28). A majority of the study participants will be admitted to a hospital ward as per standard of care for isolation of patients with smear positive pulmonary TB. Participants not considered in need of hospital admission or isolation will be followed by frequent outpatient visits as well as hospital admission on day 1 and 14 to facilitate the frequent PK-sampling. The study is registered at ClinicalTrials.gov (NCT04694586) and opened for recruitment 23rd of November 2020.

Study participants

A total number of 40 evaluable patients will be included. Study inclusion and exclusion criteria are listed in Table 1.

Table 1. Inclusion and exclusion criteria in HighShort-RP.

Inclusion criteria	*Exclusion criteria*
Adult (≥18 year)	Treated for TB within the last year.
Pulmonary TB confirmed by PCR and/or culture for *Mtb*.	Molecular or phenotypic resistance to any of the first-line oral drugs.
Eligible for first-line TB treatment, but not yet started treatment.	Miliary TB or advanced pulmonary TB (sputum smear grade 3 or advanced TB on

For peer review only - http://bmjopen.bmj.com/site/about/guidelines.xhtml

	chest X-ray according to National Tuberculosis Association, 1961, (29)).
Hiv-negative	Any other infectious disease of such significance that urgent treatment is required.
BMI (Body Mass Index) >17	Ongoing treatment with other medication where treatment with rifampicin can interact with the medication and compensatory drug adjustments cannot be made.
Written informed consent to participate in the study.	Previously known allergy to any of the first-line drugs or other contraindications according to the Summary of Product Characteristics (SmPC) of rifampicin, isoniazid, pyrazinamide, and ethambutol.
Fertile women must agree to use a barrier contraceptive during the study and have a negative pregnancy test on inclusion.	Immunocompromised defined as primary or secondary immunodeficiency including immunosuppressive treatment such as chemotherapy, immunomodulating agents, corticosteroids equivalent to ≥15 mg prednisolone.
	Known liver disease or aspartate aminotransferase (AST) or alanine aminotransferase (ALT) >1.5 times the upper normal level (UNL).
	Heart (NYHA class III and IV)
	Kidney failure (eGFR <50 mL/min)
	Dysregulated diabetes mellitus
	Alcohol or drug abuse
	Pregnancy or breast feeding
	Bodyweight <35 or >90 kg

For peer review only

Study outline

The overall study outline is shown in Figure 1.

Figure 1. The study outline in the flow-chart arrow reflects study activity identical for the intervention and control group. After four months treatment, the intervention group is followed-up according to the follow-up information found in the bottom of the flow-chart arrow, meanwhile the control group is followed-up according to routine care (not included in the picture). Study participants are provided with a drug diary to record concomitant drugs, food intake and potential side-effects during the treatment. Sputum samples are collected regularly during the study to assess time to positivity (TTP) in BACTEC MGIT 960 for mycobacterial liquid culture. Rich blood sampling for measurement of drug concentrations is collected on the day of the first dose and after 14 days of treatment. Changes in clinical status is assessed by a clinical scoring tool (TBscore II) in addition to evaluation by chest X-ray and/or thoracic computed tomography. Safety data is monitored closely during the first four study months and is based on urgently reported serious adverse events, adverse event (AE) questionnaire, medical records, regularly surveillance of blood chemistry and during the first two weeks analysis of electrocardiogram (ECG). The final treatment outcome, according to WHO definitions and occurrence of relapse, is registered for both study arms after treatment completion.

After providing informed consent patients are screened for eligibility to participate in the study based on the inclusion and exclusion criteria (Table 1). A review and registration of the patient's clinical status and symptoms is made, and any pre-existing symptoms and findings are recorded for future reference as potential adverse event (AE) from high-dose pyrazinamide and rifampicin can overlap with the symptoms of active TB. Provided all the inclusion criteria and none of the exclusion criteria are fulfilled according to the responsible physician, the study participant is electronically randomised in a 1:3 ratio to either open-label standard first-line TB treatment for six months or to a shortened treatment of four months in the intervention group. The higher doses utilised in the intervention group are 40 mg/kg of pyrazinamide (for 8 weeks) and 35 mg/kg of rifampicin (for 4 months) and with standard dosing of isoniazid (5 mg/kg for 4 months) and ethambutol (15-20 mg/kg for 8 weeks) (Table 2). Dose adjustment based on changes in body weight will be evaluated after two weeks of therapy.

Table 2. Treatment in control and intervention group.

Drug	*Control group*	*Intervention group*
Pyrazinamide	20-30 mg/kg/day for 8 weeks	40 mg/kg/day for 8 weeks

For peer review only

Rifampicin	10 mg/kg/day for 6 months	35 mg/kg/day for 4 months
Isoniazid	5 mg/kg/day for 6 months	5 mg/kg/day for 4 months
Ethambutol	15-20 mg/kg/day for 8 weeks	15-20 mg/kg/day for 8 weeks

Clinical evaluation of the participants and disease severity is assessed and documented according to TBscore II (Table 3) as well as with chest X-ray (29, 30).

Table 3. Parameters used for the TBscore II (30).

Parameters	*Points assigned*
Self-reported	
Cough	1
Dyspnoea	1
Chest pain	1
Anaemia (pale conjunctiva)	1
BMI (Body Mass Index) <18	1
BMI <16	1
MUAC (Middle Upper Arm Circumference) <220 mm	1
MUAC <200 mm	1

First-line drug concentration measurements in plasma are determined at 0, 1, 2, 4, 6, 8, 12, and 24 hrs after drug intake on day 1 and day 14. Maximal drug concentration (C_{max}) and area under the concentration-time curve ($AUC_{0\text{-}24h}$) are assessed by non-compartmental analysis using the linear trapezoidal method (31). The potential for early model-informed precision dosing of high-dose pyrazinamide and rifampicin will be explored by applying PK modelling on drug concentrations obtained after the first dose versus after 14 days of treatment. Induced sputum samples are collected on day 0, 5, 8, 15 and week 8.

For peer review only

Collection of safety measures and follow-up

All study participants are monitored throughout the study as per routine care. Additional study-specific visits and monitoring by electrocardiogram (ECG), blood sampling, sputum collection and radiology is outlined in Figure 1. Participants in the study will be provided with a diary to record drug intake and to note any suspected side-effects from the treatment. Safety data are collected during the intensive phase by weekly AE questionnaire interviews and from the patient diary, the medical record and by frequent laboratory monitoring for blood chemistry tests as displayed in Figure 1. During the continuation phase the same safety routine is applied every second week. Adverse events are graded based on Common Terminology Criteria for Adverse Events v 5.0 (CTCAE v 5.0). A Serious Adverse Event (SAE) needs to be reported within 24 hours after the study team has been notified and is defined as death of any cause, life-threatening AE and AE that results in prolonged or new hospital admission or permanent disability. For safety reasons, AE considered to be related to TB treatment and causes cessation of pyrazinamide and/or rifampicin, needs to be reported in the same manner as SAE. Any new concomitant medication is recorded and evaluated to check for potential interactions with the TB treatment. As an additional safety measure, sputum samples collected during the intensive phase will be used to evaluate the effect of combined high-dose treatment on early bactericidal activity (EBA) by analysis of time to positivity (TTP) automatically detected in BACTEC MGIT 960 (MGIT) for mycobacterial liquid culture. Adequate microbiological response is defined as sputum culture conversion between day 0 and 15 or difference in TTP between day 0 and 5 by at least 25 hours, day 0 and 8 by 50 hours or day 0 and 15 by 75 hours based on unpublished data of TTP-dynamics observed in our observational cohort study (23) and the literature (32-34).

Study participants randomised to the intervention group will be followed for a total of 28 months to record any signs of relapse. Study participants randomised to standard of care will

For peer review only

have a follow-up visit 6 to 12 months after completion of treatment and any signs of relapse beyond that point will be retrieved by data collection from the Swedish national tuberculosis registry 24 months after treatment completion. In addition to registration of relapse, final treatment outcome is recorded according to WHO definitions (35).

Bioanalytic methods

Drug concentration measurement

Blood is collected in 6 ml sodium-heparin blood tubes, centrifuged at 2000 g for 10 min within 1 hour from sampling. Aliquots of plasma is frozen in –70°C pending analysis, which will be made every three months (36, 37) at the Department of Clinical Pharmacology, Linköping University Hospital, Sweden (accredited ISO/IEC 17025:2018). Total drug concentration of isoniazid, acetyl isoniazid, rifampicin, pyrazinamide, and ethambutol will be determined by preparing samples with organic solvent protein precipitation in accordance with Fang et al and Han et al (38, 39). The samples will be analysed with liquid chromatography on a reversed phase column ACQUITY UPLC BEH C18 Column, 130Å, 1.7 µm, 2.1 mm X 50 mm a BEH C18 VanGuard pre-column and column in-line Filter (Waters Corporation, Milford, USA) and with tandem-mass spectrometry MS detection using a tandem quadrupole mass spectrometer (Xevo TQ MS, Waters) in positive electrospray ionization mode with the following quantification traces; isoniazid 137.96 > 121.04, acetyl isoniazid 180.0 > 121.0, rifampicin 823.30 > 791.416, pyrazinamide 123.95 > 54.1, and ethambutol 205.1 > 116.15. The analytic range for isoniazid is 0.25-8 mg/l, acetyl isoniazid 0.25-8 mg/l, rifampicin 2.5-80 mg/l, pyrazinamide 2.5-80 mg/l, and ethambutol 0.25-8 mg/l. The drug concentration analysis method is validated according to EMA guideline on bioanalytical method validation (40). For the quality controls at 0.75 mg/l and 6 mg/l for isoniazid, acetyl isoniazid and ethambutol the inter-day accuracy is between 95.8-99.9 % and with a precision between 7.7-10.5 % and for pyrazinamide and rifampicin quality controls at

For peer review only

233 7.5 and 60 mg/l the accuracy is between 92.3-97.6 % and precision between 9.3-11.8 %. Intra-day at 0.75 mg/l and 6 mg/l for isoniazid, acetyl isoniazid and ethambutol gave accuracy between 86.7-95.6 % and a precision of 0.6-5.2 % and for pyrazinamide and rifampicin at 7.5 and 60 mg/l an accuracy between 92.3-108.8 % and precision of 2.2-7.5 %.

237 **Microbiology**

238 *Time to positivity (TTP)*

239 Sputum samples are treated according to routine and are carried out at a biosafety level 3 laboratory at the accredited TB laboratory of the Department of Clinical Microbiology at Linköping University Hospital, Sweden. In short, following treatment of sputum with sputolysine, samples are centrifuged and resuspended in 1.5% NaOH-SDS with 2 mm glass beads and vigorously stirred using a vortex mixer. After neutralisation by 0.09% sulfuric acid samples are washed in phosphate buffer and added to MGIT-tubes according to the instructions by the manufacturer. The MGIT-tubes are incubated using BACTEC MGIT 960 (Becton Dickinson, Lakes, NJ, USA) at 37°C for up to 42 days. When growth is detected TTP is automatically recorded, *Mtb* isolates are stored at -70°C awaiting MIC determination.

248 *Minimum inhibitory concentrations (MIC)*

249 Previously stored *Mtb* isolates (-70°C) from study participant obtained before treatment initiation will be used for MIC determination of first-line drugs. MIC determination will be performed for rifampicin, isoniazid and ethambutol by broth microdilution in accordance with the EUCAST reference method for *Mtb*, and when applicable additional group A drugs as displayed in Figure 2 (41). MIC is defined as the lowest antibiotic concentration without growth when the 1:100 diluted controls show visual growth. *M. tuberculosis* H37Rv ATCC 27294 will be included as an internal quality control in each batch. Breakpoints established by the Clinical and Laboratory Standards Institute (CSLI) are used for drug susceptibility testing

For peer review only

(DST) of isolates for rifampicin, isoniazid, and ethambutol in broth microdilution plates (1, 0.12 and 8 mg/L, respectively) (42). Routine DST of first-line drugs and MIC determination of pyrazinamide are performed in MGIT as previously described with WHO-based critical concentrations of antibiotics for routine DST (rifampicin 0.5, isoniazid 0.1, pyrazinamide 100, and ethambutol 5 mg/L) and by using serial 2-fold dilutions of antibiotics for pyrazinamide MIC testing (43). Pyrazinamide will also be included in the reference method (pH 5.9) as an internal validation (Figure 2).

Figure 2. Plate outline for MIC determination by broth microdilution using the EUCAST reference method. dH_2O=distilled water, GC=growth control, RIF=rifampicin, INH=isoniazid, LEV=levofloxacin, PZA=pyrazinamide, EMB=ethambutol, LIN=linezolid

Data analysis plan

Study monitoring is performed regularly by the regional support unit for clinical trials, Forum Östergötland, at all study sites before study opening, during recruitment period and at study closure. Obtained study data are entered in electronic case record forms and managed by using REDCap electronic data capture tools (44, 45) hosted at Linköping University and at time of data analysis transferred to a database for statistical processing. The distribution of $AUC_{0\text{-}24h}$, C_{max} and PK-PD indices ($AUC_{0\text{-}24h}$/MIC, C_{max}/MIC) will be visualised separately for the two study arms. Difference in drug exposure will be explored by Mann-Whitney's U-test. A p-value of ≤ 0.05 will be considered statistically significant. The PK-PD indices based on drug exposure at day 14 for the investigational products (pyrazinamide and rifampicin) will be correlated to tentative PK-PD targets derived from the literature on human studies such as the suggested $AUC_{0\text{-}24h}$ >363 mg*h/L and AUC/MIC >8.42 for pyrazinamide (46). Associations between drug exposure and liver laboratory assessments will be analysed by using Spearman rank correlation. The incidence of minimum grade two AE and SAE will be presented in frequency tables in addition to correlation graphs on exposure-tolerability.

For peer review only

Nonlinear mixed effects modelling will be utilised to derive a population PK model for high-dose pyrazinamide. A model-informed precision dosing approach will be derived in order to be able to predict optimised dose given information from early PK sampling. An earlier developed model-informed precision dosing approach for high-dose rifampicin (47, 48) will be applied to evaluate the accuracy in the high-dose predictions. The sample size of 10 participants in the control group and 30 in the intervention group will enable sufficient descriptive PK data of the combined high-dose pyrazinamide and rifampicin regimen accounting for the expected larger PK-variability in this group compared with the control group (48). Applying EBA as one of the safety endpoints, we estimate 30% difference in EBA between the study arms (80% power, p=0.05), based on previous unpublished data of TTP-dynamics observed in our observational cohort study (23) combined with EBA data from the literature (9, 10, 49). Randomisation is performed in blocks stratified by gender and will continue until 10 individuals in the control arm and 30 individuals in the experimental arm have completed PK sampling day 14. The randomisation scheme was done with the statistical program R version 4 (the R foundation) by an independent statistician, uploaded into the CRF-program REDCap (44, 45) and blinded to the person who includes patients. Resistance to first-line drugs is rare in Sweden and MDR TB accounted for 1.8% of all new TB cases 2019 (28) and the majority of the drug resistant cases are detected early. Thus, late exclusions from the study based on detected drug resistance, are expected to be negligible.

Patient and public involvement

Patients were not involved in the development of the research plan nor the implementation of this clinical trial. We aim to present study results at a conference where patient representatives or patient communities are present. Furthermore, patients will be involved in a future phase III trial based on the results of this study.

Ethics and dissemination

For peer review only

This study has been approved by the Swedish Ethical Review Authority (approval number 2020-00761) and the Swedish Medical Products Agency (approval number 5.1-2019-105207 and EudraCT number 2019-003721-25). Prior to the study opening, a designated study team of nurses, physiotherapists, laboratory staff and clinical doctors participated in training sessions of the study protocol which included study-specific procedures and ethical considerations. Study initiation meeting and the main training sessions were led by the coordinating principal investigator assisted by the support unit for clinical trials Forum Östergötland. Physicians and nurses in the study team have documented training in Good Clinical Practice (GCP) and the study is performed in accordance with GCP and the Declaration of Helsinki. Informed consent is obtained from each study participant by a physician prior to any study-specific activity. Inclusion of patients in the study is restricted to the available set of written informed consent translated into selected languages. In addition and when required, an interpreter will be used for providing study information and to translate during study visits. Study participants are free to withdraw from the study at any time and will at that point be offered treatment according to standard of care. The safety of combined high-dose pyrazinamide (40 mg/kg) and rifampicin (35 mg/kg) has not been studied in humans before. However, TB treatment with rifampicin 35 mg/kg given to 63 patients for 12 weeks has been proven safe (10). Furthermore, data was recently presented on exposure-tolerability of two weeks treatment with rifampicin 40 mg/kg (n=15) and 50 mg/kg (n=17) where the dose of 40 mg/kg was safe and tolerable (25). In early studies on lengthy treatments of pyrazinamide beyond 8 weeks, increased liver markers, jaundice and arthralgia were associated with higher doses of pyrazinamide (50). In a meta-analysis comparing rates of AEs between different doses of pyrazinamide, the frequencies of hepatotoxicity were 0.042 (95% CI, 0.026 to 0.067) for 30 mg/kg, 0.055 (95% CI, 0.031 to 0.094) for 40 mg/kg, and 0.098 (95% CI, 0.047 to 0.193) for 60 mg/kg, suggesting a considerable proportion of

For peer review only - http://bmjopen.bmj.com/site/about/guidelines.xhtml

hepatoxicity rates may be idiosyncratic rather than dose-dependent (51). According to a meta-analysis (n=4490), liver toxicity did not seem to be dose-dependent until pyrazinamide exposures exceeded a weekly AUC of 5000 mg*h/L (52) compared to an AUC of 363 mg*h/L on standard dosing (46). Recently, a patient-level data analysis where patients received rifampicin (range 10-35 mg/kg), pyrazinamide (range 20-30 mg/kg), plus two companion drugs, found liver toxicity to be rare (3.9% with Grade 3 or higher liver function tests, LFT), and no relationship was observed between pyrazinamide C_{max} and LFT levels (24). Furthermore, currently used doses of 30-40 mg/kg pyrazinamide daily as part of MDR-TB treatment are well-tolerated (53). In summary, we consider it safe for patients to participate in our clinical trial and study participants will be closely monitored regarding potential side effects including weekly measurement of LFT during the first 8 weeks when high-dose pyrazinamide and rifampicin are combined.

Regardless of study results, we plan to present our data in international conferences and to publish our results in a peer-reviewed journal. Any significant protocol amendments will be reported to the ethical review authority and medical products agency.

DISCUSSION

There is strong urge for a shortened treatment of drug-susceptible TB. In this phase II clinical trial (HighShort-RP) of a shorter experimental TB regimen based on high-dose pyrazinamide and rifampicin, tolerability and safety will be investigated along with in-depth PK-PD analysis including determination of individual MICs. In a PK-PD modelling study published just after our study opened for recruitment, the relationship between pyrazinamide exposures and efficacy or hepatotoxicity was assessed on pooled retrospective data from three clinical interventional trials conducted by the Tuberculosis Trials Consortium and Pan African Consortium for the Evaluation of Antituberculosis Antibiotics Networks (24). Simulations

For peer review only - http://bmjopen.bmj.com/site/about/guidelines.xhtml

identified two potentially treatment-shortening strategies restricted to currently used first-line drugs whereof one was to optimise drug exposure of rifampicin and pyrazinamide in parallel. The authors stated that whether or not an enhanced multidrug regimen based on high-dose rifampicin (e.g. ≥35 mg/kg) and higher-dose pyrazinamide (e.g. 30-40 mg/kg) will be sufficient to significantly reduce the length of TB treatment must be explored prospectively, with attention to safety and tolerability. Results from our clinical trial will be indicative whether the combined high-dose rifampicin (35 mg/kg) with pyrazinamide (40 mg/kg) along with standard doses of isoniazid and ethambutol, is tolerable for patients. Furthermore, the PK-PD data retrieved from the study can be included in predictions of effective treatment-shortening regimens launched in larger RCTs evaluating high-dose sterilising regimens with non-inferiority criteria compared with standard of care. Recently, a large RCT of pulmonary TB patients showed that the efficacy of a 4-month rifapentine-based regimen containing moxifloxacin was noninferior to standard 6-month TB regimen in terms of TB disease-free survival 12 months after randomisation (54). Our clinical trial aim to achieve maximum output of the first-line drugs. A fluoroquinolone-based treatment-shortening regimen for drug-susceptible TB would result in distributing this important drug to many more people, which in turn carries the risk of increased global acquired fluoroquinolone resistance such as additional resistance in MDR TB isolates (increased number of pre-XDR cases) and in other bacterial pathogens e.g., gram-negative bacteria.

Personalised medicine based on disease severity and dose-adjustments guided by therapeutic drug monitoring to achieve individual maximum exposures of TB drugs is promising, in particularly as the variation of drug exposures is extensive during high-dose rifampicin treatment (9, 14). In addition, combined treatment of high-dose rifampicin and pyrazinamide is likely to affect the variation of pyrazinamide exposure as pyrazinamide clearance has been reported to increase 15-30% in a rifampicin-containing regimen probably due to the

For peer review only

autoinduction of the CYP450-system mediated by rifampicin (55, 56). This highlights the value of measuring drug exposure. In addition, the prerequisites for early model-informed precision dosing based on PK data obtained after the first dose will be explored in this phase II clinical trial.

Data sharing statement

No data are currently available.

Author's contribution

The study design was developed within the TB research group at Linköping University Hospital led by JP and KN with support from JWA and USHS on the pharmacokinetic part of the study. Statistician MF assisted in statistical selection of study dimensions and developed the block randomisation procedure. AB, JP and KN wrote the study protocol and the applications to ethical review authority and medical product agency. KN is the coordinating principal investigator of the clinical trial and research nurse in lead is MS. KN developed case record forms (CRFs), standard operating procedures (SOPs), led study initiation meeting with training sessions together with DE and assisted by DN, MS, MJN, MSa and JP. BC is the contact person at the laboratory of the Department of Clinical Pharmacology and MJN at the TB laboratory of the Department of Clinical Microbiology. DE and KN wrote the first draft of the manuscript. All authors revised and approved the final version of the manuscript.

Funding statements

This work was supported by the Swedish National Research Council (KN, grant number 2019-05912), the Swedish Heart Lung Foundation (JP, grant number 20200302), the Research Council of south-eastern Sweden (JP, grant number FORSS-931028) and the Region Östergötland ALF grant (JP, grant number RÖ-888331). The funders had no role in any of the stages of the study.

Competing interest

None to declare.

Acknowledgments

We thank all members of the study team at the Infectious Diseases Clinic of Östergötland, former research nurse Lisa Gunnarsson at the Department of Infectious Diseases at Linköping University Hospital, initiated local principal investigators and the research coordinator Lina Malm at Forum Östergötland, Linköping University.

For peer review only

REFERENCES

1. World Health Organization. Guidelines for treatment of drug-susceptible tuberculosis and patient care (2017 update). Available from: https://www.who.int/tb/publications/2017/dstb_guidance_2017/en/.
2. Controlled clinical trial of short-course (6-month) regimens of chemotherapy for treatment of pulmonary tuberculosis. Lancet. 1972;1(7760):1079-85.
3. Fox W, Ellard GA, Mitchison DA. Studies on the treatment of tuberculosis undertaken by the British Medical Research Council tuberculosis units, 1946-1986, with relevant subsequent publications. Int J Tuberc Lung Dis. 1999;3(10 Suppl 2):S231-79.
4. Merle CS, Fielding K, Sow OB, Gninafon M, Lo MB, Mthiyane T, et al. A four-month gatifloxacin-containing regimen for treating tuberculosis. N Engl J Med. 2014;371(17):1588-98.
5. Gillespie SH, Crook AM, McHugh TD, Mendel CM, Meredith SK, Murray SR, et al. Four-month moxifloxacin-based regimens for drug-sensitive tuberculosis. N Engl J Med. 2014;371(17):1577-87.
6. Jawahar MS, Banurekha VV, Paramasivan CN, Rahman F, Ramachandran R, Venkatesan P, et al. Randomized clinical trial of thrice-weekly 4-month moxifloxacin or gatifloxacin containing regimens in the treatment of new sputum positive pulmonary tuberculosis patients. PLoS One. 2013;8(7):e67030.
7. Jindani A, Harrison TS, Nunn AJ, Phillips PP, Churchyard GJ, Charalambous S, et al. High-dose rifapentine with moxifloxacin for pulmonary tuberculosis. N Engl J Med. 2014;371(17):1599-608.
8. Imperial MZ, Nahid P, Phillips PPJ, Davies GR, Fielding K, Hanna D, et al. A patient-level pooled analysis of treatment-shortening regimens for drug-susceptible pulmonary tuberculosis. Nat Med. 2018;24(11):1708-15.
9. Boeree MJ, Diacon AH, Dawson R, Narunsky K, du Bois J, Venter A, et al. A dose-ranging trial to optimize the dose of rifampin in the treatment of tuberculosis. Am J Respir Crit Care Med. 2015;191(9):1058-65.
10. Boeree MJ, Heinrich N, Aarnoutse R, Diacon AH, Dawson R, Rehal S, et al. High-dose rifampicin, moxifloxacin, and SQ109 for treating tuberculosis: a multi-arm, multi-stage randomised controlled trial. Lancet Infect Dis. 2017;17(1):39-49.
11. Aarnoutse RE, Kibiki GS, Reither K, Semvua HH, Haraka F, Mtabho CM, et al. Pharmacokinetics, Tolerability, and Bacteriological Response of Rifampin Administered at 600, 900, and 1,200 Milligrams Daily in Patients with Pulmonary Tuberculosis. Antimicrob Agents Chemother. 2017;61(11).
12. Velasquez GE, Brooks MB, Coit JM, Pertinez H, Vargas Vasquez D, Sanchez Garavito E, et al. Efficacy and Safety of High-Dose Rifampin in Pulmonary Tuberculosis. A Randomized Controlled Trial. Am J Respir Crit Care Med. 2018;198(5):657-66.
13. Svensson EM, Svensson RJ, Te Brake LHM, Boeree MJ, Heinrich N, Konsten S, et al. The Potential for Treatment Shortening With Higher Rifampicin Doses: Relating Drug Exposure to Treatment Response in Patients With Pulmonary Tuberculosis. Clin Infect Dis. 2018;67(1):34-41.
14. Svensson RJ, Svensson EM, Aarnoutse RE, Diacon AH, Dawson R, Gillespie SH, et al. Greater Early Bactericidal Activity at Higher Rifampicin Doses Revealed by Modeling and Clinical Trial Simulations. J Infect Dis. 2018;218(6):991-9.
15. Zhang Y, Shi W, Zhang W, Mitchison D. Mechanisms of Pyrazinamide Action and Resistance. Microbiol Spectr. 2014;2(4):Mgm2-0023-2013.
16. Controlled trial of 6-month and 8-month regimens in the treatment of pulmonary tuberculosis. First report. Am Rev Respir Dis. 1978;118(2):219-28.

For peer review only

17. Clinical trial of six-month and four-month regimens of chemotherapy in the treatment of pulmonary tuberculosis: the results up to 30 months. Tubercle. 1981;62(2):95-102.
18. Chideya S, Winston CA, Peloquin CA, Bradford WZ, Hopewell PC, Wells CD, et al. Isoniazid, rifampin, ethambutol, and pyrazinamide pharmacokinetics and treatment outcomes among a predominantly HIV-infected cohort of adults with tuberculosis from Botswana. Clin Infect Dis. 2009;48(12):1685-94.
19. Pasipanodya JG, McIlleron H, Burger A, Wash PA, Smith P, Gumbo T. Serum drug concentrations predictive of pulmonary tuberculosis outcomes. J Infect Dis. 2013;208(9):1464-73.
20. Perumal R, Naidoo K, Naidoo A, Ramachandran G, Requena-Mendez A, Sekaggya-Wiltshire C, et al. A systematic review and meta-analysis of first-line tuberculosis drug concentrations and treatment outcomes. Int J Tuberc Lung Dis. 2020;24(1):48-64.
21. Alsultan A, Peloquin CA. Therapeutic drug monitoring in the treatment of tuberculosis: an update. Drugs. 2014;74(8):839-54.
22. Rockwood N, Pasipanodya JG, Denti P, Sirgel F, Lesosky M, Gumbo T, et al. Concentration-Dependent Antagonism and Culture Conversion in Pulmonary Tuberculosis. Clin Infect Dis. 2017;64(10):1350-9.
23. Niward K, Davies Forsman L, Bruchfeld J, Chryssanthou E, Carlstrom O, Alomari T, et al. Distribution of plasma concentrations of first-line anti-TB drugs and individual MICs: a prospective cohort study in a low endemic setting. J Antimicrob Chemother. 2018;73(10):2838-45.
24. Zhang N, Savic RM, Boeree MJ, Peloquin C, Weiner M, Heinrich N, et al. Optimising pyrazinamide for the treatment of tuberculosis. Eur Respir J. 2021.
25. Te Brake LHM, De Jager V, Narunsky K, Vanker N, Svensson EM, Phillips PPJ, et al. Increased bactericidal activity but dose-limiting intolerability at 50 mg·kg−1 rifampicin. European Respiratory Journal. 2021:2000955.
26. Chan AW, Tetzlaff JM, Altman DG, Laupacis A, Gøtzsche PC, Krleža-Jerić K, et al. SPIRIT 2013 statement: defining standard protocol items for clinical trials. Ann Intern Med. 2013;158(3):200-7.
27. Chan AW, Tetzlaff JM, Gøtzsche PC, Altman DG, Mann H, Berlin JA, et al. SPIRIT 2013 explanation and elaboration: guidance for protocols of clinical trials. Bmj. 2013;346:e7586.
28. Folkhälsomyndigheten. Tuberkulos 2019 – utökad version. Available from: https://www.folkhalsomyndigheten.se/folkhalsorapportering-statistik/statistik-a-o/sjukdomsstatistik/tuberkulos/arsrapporter-och-kommentarer/2019/2019/ Accessed 2021-05-13.
29. American Thoracic Society. National Tuberculosis Association of the USA. Diagnostic Standards and Classification of Tuberculosis New York, NY, USA: National Tuberculosis Association; 1961.
30. Rudolf F, Lemvik G, Abate E, Verkuilen J, Schon T, Gomes VF, et al. TBscore II: refining and validating a simple clinical score for treatment monitoring of patients with pulmonary tuberculosis. Scand J Infect Dis. 2013;45(11):825-36.
31. Rowland M, Tozer TN. Clinical pharmacokinetics and pharmacodynamics; Concepts and applications, 4th ed. Philadelphia USA: Lippincott Williams and Wilkins; 2011.
32. Diacon AH, Dawson R, von Groote-Bidlingmaier F, Symons G, Venter A, Donald PR, et al. 14-day bactericidal activity of PA-824, bedaquiline, pyrazinamide, and moxifloxacin combinations: a randomised trial. The Lancet. 2012;380(9846):986-93.
33. Diacon A, Miyahara S, Dawson R, Sun X, Hogg E, Donahue K, et al. Assessing whether isoniazid is essential during the first 14 days of tuberculosis therapy: a phase 2a, open-label, randomised controlled trial. The Lancet Microbe. 2020;1(2):e84-e92.
34. Sabiiti W, Azam K, Farmer ECW, Kuchaka D, Mtafya B, Bowness R, et al. Tuberculosis bacillary load, an early marker of disease severity: the utility of tuberculosis Molecular Bacterial Load Assay. Thorax. 2020;75(7):606-8.

For peer review only

35. World Health Organization. Definitions and reporting framework for tuberculosis 2013 revision, updated December 2014, Geneva: World Health Organization Available from: https://www.who.int/tb/publications/definitions/en/.

36. Le Guellec C, Gaudet ML, Lamanetre S, Breteau M. Stability of rifampin in plasma: consequences for therapeutic monitoring and pharmacokinetic studies. Ther Drug Monit. 1997;19(6):669-74.

37. Zhou Z, Wu X, Wei Q, Liu Y, Liu P, Ma A, et al. Development and validation of a hydrophilic interaction liquid chromatography–tandem mass spectrometry method for the simultaneous determination of five first-line antituberculosis drugs in plasma. Analytical and Bioanalytical Chemistry. 2013;405(19):6323-35.

38. Fang P-F, Cai H-L, Li H-D, Zhu R-H, Tan Q-Y, Gao W, et al. Simultaneous determination of isoniazid, rifampicin, levofloxacin in mouse tissues and plasma by high performance liquid chromatography–tandem mass spectrometry. Journal of Chromatography B. 2010;878(24):2286-91.

39. Han M, Jun SH, Lee JH, Park KU, Song J, Song SH. Method for simultaneous analysis of nine second-line anti-tuberculosis drugs using UPLC-MS/MS. Journal of Antimicrobial Chemotherapy. 2013;68(9):2066-73.

40. European Medicines Agency. Guideline on bioanalytical method validation, 21 July 2011, 192217.

41. Schön T, Werngren J, Machado D, Borroni E, Wijkander M, Lina G, et al. Antimicrobial susceptibility testing of Mycobacterium tuberculosis complex isolates - the EUCAST broth microdilution reference method for MIC determination. Clin Microbiol Infect. 2020;26(11):1488-92.

42. CLSI. Performance Standards for Susceptibility Testing of Mycobacteria, Nocardia spp., and Other Aerobic Actinomycetes. 1st ed. CLSI supplement. Wayne, PA: Clinical and Laboratory Standards Institute; 2018.

43. Sturegard E, Angeby KA, Werngren J, Jureen P, Kronvall G, Giske CG, et al. Little difference between minimum inhibitory concentrations of Mycobacterium tuberculosis wild-type organisms determined with BACTEC MGIT 960 and Middlebrook 7H10. Clin Microbiol Infect. 2015;21(2):148.e5-7.

44. Harris PA, Taylor R, Thielke R, Payne J, Gonzalez N, Conde JG. Research electronic data capture (REDCap)—A metadata-driven methodology and workflow process for providing translational research informatics support. Journal of Biomedical Informatics. 2009;42(2):377-81.

45. Harris PA, Taylor R, Minor BL, Elliott V, Fernandez M, O'Neal L, et al. The REDCap consortium: Building an international community of software platform partners. Journal of Biomedical Informatics. 2019;95:103208.

46. Sturkenboom MGG, Märtson AG, Svensson EM, Sloan DJ, Dooley KE, van den Elsen SHJ, et al. Population Pharmacokinetics and Bayesian Dose Adjustment to Advance TDM of Anti-TB Drugs. Clin Pharmacokinet. 2021:1-26.

47. Svensson RJ, Niward K, Davies Forsman L, Bruchfeld J, Paues J, Eliasson E, et al. Individualised dosing algorithm and personalised treatment of high-dose rifampicin for tuberculosis. Br J Clin Pharmacol. 2019;85(10):2341-50.

48. Svensson RJ, Aarnoutse RE, Diacon AH, Dawson R, Gillespie SH, Boeree MJ, et al. A Population Pharmacokinetic Model Incorporating Saturable Pharmacokinetics and Autoinduction for High Rifampicin Doses. Clin Pharmacol Ther. 2018;103(4):674-83.

49. Jindani A, Dore CJ, Mitchison DA. Bactericidal and sterilizing activities of antituberculosis drugs during the first 14 days. Am J Respir Crit Care Med. 2003;167(10):1348-54.

50. Girling DJ. The hepatic toxicity of antituberculosis regimens containing isoniazid, rifampicin and pyrazinamide. Tubercle. 1978;59(1):13-32.

For peer review only

51. Pasipanodya JG, Gumbo T. Clinical and toxicodynamic evidence that high-dose pyrazinamide is not more hepatotoxic than the low doses currently used. Antimicrob Agents Chemother. 2010;54(7):2847-54.

52. Sahota T, Della Pasqua O. Feasibility of a fixed-dose regimen of pyrazinamide and its impact on systemic drug exposure and liver safety in patients with tuberculosis. Antimicrob Agents Chemother. 2012;56(11):5442-9.

53. Borisov S, Danila E, Maryandyshev A, Dalcolmo M, Miliauskas S, Kuksa L, et al. Surveillance of adverse events in the treatment of drug-resistant tuberculosis: first global report. Eur Respir J. 2019;54(6).

54. Dorman SE, Nahid P, Kurbatova EV, Phillips PPJ, Bryant K, Dooley KE, et al. Four-Month Rifapentine Regimens with or without Moxifloxacin for Tuberculosis. New England Journal of Medicine. 2021;384(18):1705-18.

55. Denti P, Jeremiah K, Chigutsa E, Faurholt-Jepsen D, PrayGod G, Range N, et al. Pharmacokinetics of Isoniazid, Pyrazinamide, and Ethambutol in Newly Diagnosed Pulmonary TB Patients in Tanzania. PLoS One. 2015;10(10):e0141002.

56. Chirehwa MT, McIlleron H, Rustomjee R, Mthiyane T, Onyebujoh P, Smith P, et al. Pharmacokinetics of Pyrazinamide and Optimal Dosing Regimens for Drug-Sensitive and -Resistant Tuberculosis. Antimicrob Agents Chemother. 2017;61(8).

For peer review only

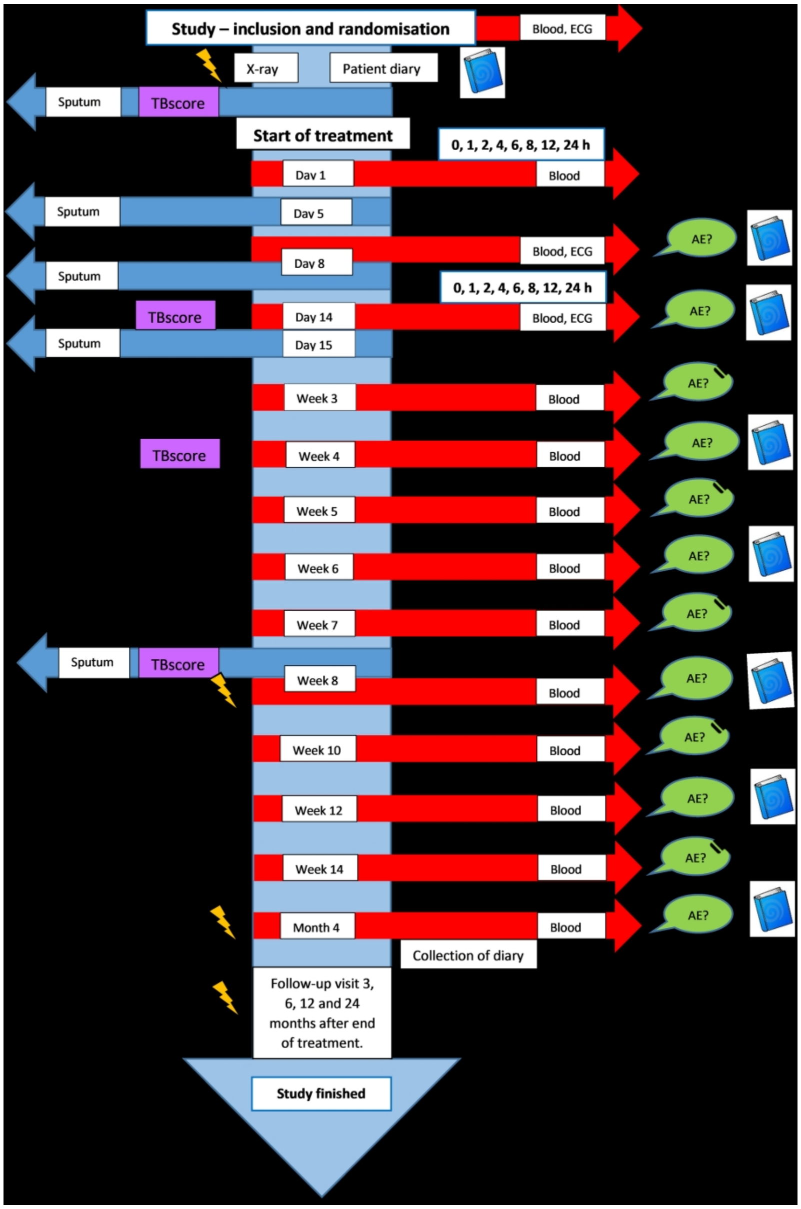

Figure 1. The study outline in the flow-chart arrow reflects study activity identical for the intervention and control group. After four months treatment, the intervention group is followed-up according to the follow-up information found in the bottom of the flow-chart arrow, meanwhile the control group is followed-up according to routine care (not included in the picture). Study participants are provided with a drug diary to record concomitant drugs, food intake and potential side-effects during the treatment. Sputum samples are collected regularly during the study to assess time to positivity (TTP) in BACTEC MGIT 960 for mycobacterial liquid culture. Rich blood sampling for measurement of drug concentrations is collected on the day of the first dose and after 14 days of treatment. Changes in clinical status is assessed by a clinical scoring tool (TBscore II) in addition to evaluation by chest X-ray and/or thoracic computed tomography. Safety data is monitored closely during the first four study months and is based on urgently reported serious adverse events, adverse event (AE) questionnaire, medical records, regularly surveillance of blood chemistry and during the first two weeks analysis of electrocardiogram (ECG). The final treatment outcome, according to WHO definitions and occurrence of relapse, is registered for both study arms after treatment completion.

159x240mm (300 x 300 DPI)

	1	2	3	4	5	6	7	8	9	10	11	12
A	200ul dH_2O	200ul dH_2O	200ul dH_2O	200ul dH_2O	200ul dH_2O	200ul dH_2O	200ul dH_2O	200ul dH_2O	200ul dH_2O	200ul dH_2O	200ul dH_2O	200ul dH_2O
B	negative control	GC 100%	RIF 0.032	RIF 0.064	RIF 0.125	RIF 0.25	RIF 0.5	RIF 1	RIF 2	RIF 4	GC 0.1%	200ul dH_2O
C	negative control	GC 100%	INH 0.016	INH 0.032	INH 0.06	INH 0.125	INH 0.25	INH 0.5	INH 1	INH 2	GC 1%	200ul dH_2O
D	negative control	GC 100%	LEV 0,03	LEV 0,06	LEV 0,12	LEV 0,25	LEV 0,5	LEV 1	LEV 2	LEV 4	GC 1%	200ul dH_2O
E	negative control	GC 1%	PZA 2	PZA 4	PZA 8	PZA 16	PZA 32	PZA 64	PZA 128	PZA 256	GC 10%	200ul dH_2O
F	negative control	GC 1%	EMB 0.125	EMB 0.25	EMB 0.5	EMB 1	EMB 2	EMB 4	EMB 8	EMB 16	GC 100%	200ul dH_2O
G	negative control	GC 1%	LIN 0.064	LIN 0.125	LIN 0.25	LIN 0.5	LIN 1	LIN 2	LIN 4	LIN 8	GC 100%	200ul dH_2O
H	200ul dH_2O	200ul dH_2O	200ul dH_2O	200ul dH_2O	200ul dH_2O	200ul dH_2O	200ul dH_2O	200ul dH_2O	200ul dH_2O	200ul dH_2O	200ul dH_2O	200ul dH_2O

Figure 2. Plate outline for MIC determination by broth microdilution using the EUCAST reference method. dH2O=distilled water, GC=growth control, RIF=rifampicin, INH=isoniazid, LEV=levofloxacin, PZA=pyrazinamide, EMB=ethambutol, LIN=linezolid

630x354mm (236 x 236 DPI)

SPIRIT Checklist for *Trials*

Complete this checklist by entering the page and line numbers where each of the items listed below can be found in your manuscript.

Your manuscript may not currently address all the items on the checklist. Please modify your text to include the missing information. If you are certain that an item does not apply, please state "n/a" and provide a short explanation. **Leaving an item blank or stating "n/a" without an explanation will lead to your manuscript being returned before review.**

Upload your completed checklist as an additional file when you submit to *Trials*. You must reference this additional file in the main text of your protocol submission. The completed SPIRIT figure must be included within the main body of the protocol text and can be downloaded here: http://www.spirit-statement.org/schedule-of-enrolment-interventions-and-assessments/

In your methods section, please state that you used the SPIRIT reporting guidelines, and cite them as:

Chan A-W, Tetzlaff JM, Gøtzsche PC, Altman DG, Mann H, Berlin J, Dickersin K, Hróbjartsson A, Schulz KF, Parulekar WR, Krleža-Jerić K, Laupacis A, Moher D. SPIRIT 2013 Explanation and Elaboration: Guidance for protocols of clinical trials. BMJ. 2013;346:e7586

		Reporting Item	Page and Line Number	Reason if not applicable
**Administrative information**				
Title	#1	Descriptive title identifying the study design, population, interventions, and, if applicable, trial acronym	Line 1-4	
Trial registration	#2a	Trial identifier and registry name. If not yet registered, name of intended registry	Line 57	
Trial registration: data set	#2b	All items from the World Health Organization Trial Registration Data Set	Line 57	Information found at ClinicalTrial.gov and in the Swedish Study Protocol currently only available in Swedish, which is sufficient for the conduct of the clinical trial. However, we intent to translate the

For peer review only

				protocol to English but due to covid-19 the process is delayed.
Protocol version	#3	Date and version identifier		Not included in manuscript but in the Swedish Study Protocol
Funding	#4	Sources and types of financial, material, and other support	Line 401-405	
Roles and responsibilities: contributorship	#5a	Names, affiliations, and roles of protocol contributors	Line 389-399	More details are included in the Swedish Study Protocol approved by the Swedish Ethical Review Authority and the Swedish Medical Products Agency.
Roles and responsibilities: sponsor contact information	#5b	Name and contact information for the trial sponsor		Not included in manuscript but in the Swedish Study Protocol. Academic investigator-initiated trial (no pharmaceutical company involved).
Roles and responsibilities: sponsor and funder	#5c	Role of study sponsor and funders, if any, in study design; collection, management, analysis, and interpretation of data; writing of the report; and the decision to submit the report for publication, including whether they will have ultimate authority over any of these activities	Line 404-405	
Roles and responsibilities: committees	#5d	Composition, roles, and responsibilities of the coordinating centre, steering committee, endpoint adjudication committee, data management team, and other individuals or groups overseeing the trial, if applicable (see Item 21a for data monitoring committee)	Line 269-271	The study has no special committees as it is not required for this type of Phase II trial according to the Swedish Medical Products Agency.

For peer review only

**Introduction**			Line 75-117	
Background and rationale	#6a	Description of research question and justification for undertaking the trial, including summary of relevant studies (published and unpublished) examining benefits and harms for each intervention	Line 80-114 and line 322-344	
Background and rationale: choice of comparators	#6b	Explanation for choice of comparators	Line 287-294	Included in "Material and analysis – Data analysis plan".
Objectives	#7	Specific objectives or hypotheses	Line 115-117	
Trial design	#8	Description of trial design including type of trial (eg, parallel group, crossover, factorial, single group), allocation ratio, and framework (eg, superiority, equivalence, non-inferiority, exploratory)	Line 120	To adhere to word limitation and avoid repetition this is declared once in the first lines of the section "Methods and analysis".
**Methods: Participants, interventions, and outcomes**				
Study setting	#9	Description of study settings (eg, community clinic, academic hospital) and list of countries where data will be collected. Reference to where list of study sites can be obtained	Line 126-137	
Eligibility criteria	#10	Inclusion and exclusion criteria for participants. If applicable, eligibility criteria for study centres and individuals who will perform the interventions (eg, surgeons, psychotherapists)	Line 139-140, Table 1	

For peer review only

Interventions: description	#11a	Interventions for each group with sufficient detail to allow replication, including how and when they will be administered	Line 159-171, Table 2	More details are included in the Swedish Study Protocol.
Interventions: modifications	#11b	Criteria for discontinuing or modifying allocated interventions for a given trial participant (eg, drug dose change in response to harms, participant request, or improving / worsening disease)	Line 184-205	Not included in detail in the manuscript but in the Swedish Study Protocol.
Interventions: adherance	#11c	Strategies to improve adherence to intervention protocols, and any procedures for monitoring adherence (eg, drug tablet return; laboratory tests)	Figure 1	Not included in detail in the manuscript but in the Swedish Study Protocol.
Interventions: concomitant care	#11d	Relevant concomitant care and interventions that are permitted or prohibited during the trial	Figure 1, line 173-174 and line 184-191	Not included in detail in the manuscript but in the Swedish Study Protocol.
Outcomes	#12	Primary, secondary, and other outcomes, including the specific measurement variable (eg, systolic blood pressure), analysis metric (eg, change from baseline, final value, time to event), method of aggregation (eg, median, proportion), and time point for each outcome. Explanation of the clinical relevance of chosen efficacy and harm outcomes is strongly recommended	122-124	
Participant timeline	#13	Time schedule of enrolment, interventions (including any run-ins and washouts), assessments, and visits for participants. A schematic diagram is highly recommended (see Figure)	Figure 1	More detailed flowchart is available for the study team in the Swedish Study Protocol approved by the Swedish Ethical Review Authority and the Swedish Medical Product Agency.

For peer review only

Sample size	#14	Estimated number of participants needed to achieve study objectives and how it was determined, including clinical and statistical assumptions supporting any sample size calculations	Line 287-296	
Recruitment	#15	Strategies for achieving adequate participant enrolment to reach target sample size	Line 126-131 and line 294-296	
**Methods: Assignment of interventions (for controlled trials)**				
Allocation: sequence generation	#16a	Method of generating the allocation sequence (eg, computer-generated random numbers), and list of any factors for stratification. To reduce predictability of a random sequence, details of any planned restriction (eg, blocking) should be provided in a separate document that is unavailable to those who enrol participants or assign interventions	Line 294-298	
Allocation concealment mechanism	#16b	Mechanism of implementing the allocation sequence (eg, central telephone; sequentially numbered, opaque, sealed envelopes), describing any steps to conceal the sequence until interventions are assigned	Line 163-167 and line 296-298	
Allocation: implementation	#16c	Who will generate the allocation sequence, who will enrol participants, and who will assign participants to interventions	Line 163-167	Not included in detail in the manuscript but in the Swedish Study Protocol.

For peer review only

Blinding (masking)	#17a	Who will be blinded after assignment to interventions (eg, trial participants, care providers, outcome assessors, data analysts), and how	Line 120 and line 165-167	The study is an academic investigator-initiated open-label phase II trial with no blinding after assignment to interventions.
Blinding (masking): emergency unblinding	#17b	If blinded, circumstances under which unblinding is permissible, and procedure for revealing a participant's allocated intervention during the trial		Not applicable.
**Methods: Data collection, management, and analysis**				
Data collection plan	#18a	Plans for assessment and collection of outcome, baseline, and other trial data, including any related processes to promote data quality (eg, duplicate measurements, training of assessors) and a description of study instruments (eg, questionnaires, laboratory tests) along with their reliability and validity, if known. Reference to where data collection forms can be found, if not in the protocol	Figure 1, line 159-163, line 173-211, Table 3 and line 271-273.	Not included in detail in the manuscript but in the Swedish Study Protocol.
Data collection plan: retention	#18b	Plans to promote participant retention and complete follow-up, including list of any outcome data to be collected for participants who discontinue or deviate from intervention protocols	Line 206-211 and line 294-296.	Lost to follow-up for patients with TB in Sweden is extremely rare. In addition, we have access to the Swedish national tuberculosis registry that keeps track on long-term outcome of every active case. More details are included in the Swedish Study Protocol.
Data management	#19	Plans for data entry, coding, security, and storage, including any related processes to		Not included in detail in the manuscript but in the Swedish Study Protocol

For peer review only

		promote data quality (eg, double data entry; range checks for data values). Reference to where details of data management procedures can be found, if not in the protocol		approved by the Swedish Ethical Review Authority and the Swedish Medical Products Agency.
Statistics: outcomes	#20a	Statistical methods for analysing primary and secondary outcomes. Reference to where other details of the statistical analysis plan can be found, if not in the protocol	Line 273-287	
Statistics: additional analyses	#20b	Methods for any additional analyses (eg, subgroup and adjusted analyses)		Not applicable for this phase II trial.
Statistics: analysis population and missing data	#20c	Definition of analysis population relating to protocol non-adherence (eg, as randomised analysis), and any statistical methods to handle missing data (eg, multiple imputation)	Line 294-296	Not included in detail in the manuscript but in the Swedish Study Protocol.
**Methods: Monitoring**				
Data monitoring: formal committee	#21a	Composition of data monitoring committee (DMC); summary of its role and reporting structure; statement of whether it is independent from the sponsor and competing interests; and reference to where further details about its charter can be found, if not in the protocol. Alternatively, an explanation of why a DMC is not needed	Line 269-271 and line 313-317	The support unit for clinical trials, Forum Östergötland, Linköping University is the independent study monitoring unit that will ensure adherence to the Study Protocol (including GCP and Declaration of Helsinki) throughout the study.
Data monitoring: interim analysis	#21b	Description of any interim analyses and stopping guidelines, including who will have access to these interim results and make the final decision to terminate the trial		Not applicable, interim analysis is not needed. More details are included in the Swedish Study Protocol regarding criteria for the sponsor to stop the study.

For peer review only

Harms	#22	Plans for collecting, assessing, reporting, and managing solicited and spontaneously reported adverse events and other unintended effects of trial interventions or trial conduct	Line 184-211 and line 346-347	
Auditing	#23	Frequency and procedures for auditing trial conduct, if any, and whether the process will be independent from investigators and the sponsor		When the Swedish Medical Product Agency approves an interventional clinical trial, audits may be performed by them at any time. Details included in the Swedish Study Protocol.
**Ethics and dissemination**				
Research ethics approval	#24	Plans for seeking research ethics committee / institutional review board (REC / IRB) approval	Line 54-55 and line 308-310	
Protocol amendments	#25	Plans for communicating important protocol modifications (eg, changes to eligibility criteria, outcomes, analyses) to relevant parties (eg, investigators, REC / IRBs, trial participants, trial registries, journals, regulators)	Line 346-347	
Consent or assent	#26a	Who will obtain informed consent or assent from potential trial participants or authorised surrogates, and how (see Item 32)	Line 317-318	
Consent or assent: ancillary studies	#26b	Additional consent provisions for collection and use of participant data and biological specimens in ancillary studies, if applicable		Not included in the manuscript but is described in patient study information/informed consent.
Confidentiality	#27	How personal information about potential and enrolled participants will be collected, shared,		Not included in the manuscript but is described in patient study information/informed consent.

For peer review only

		and maintained in order to protect confidentiality before, during, and after the trial		
Declaration of interests	#28	Financial and other competing interests for principal investigators for the overall trial and each study site	Line 406-407	
Data access	#29	Statement of who will have access to the final trial dataset, and disclosure of contractual agreements that limit such access for investigators		Not included in detail in the manuscript but in the Swedish Study Protocol.
Ancillary and post trial care	#30	Provisions, if any, for ancillary and post-trial care, and for compensation to those who suffer harm from trial participation		Not included in detail in the manuscript but in the Swedish Study Protocol and the patient study information/informed consent.
Dissemination policy: trial results	#31a	Plans for investigators and sponsor to communicate trial results to participants, healthcare professionals, the public, and other relevant groups (eg, via publication, reporting in results databases, or other data sharing arrangements), including any publication restrictions	Line 345-346 and line 304-305	
Dissemination policy: authorship	#31b	Authorship eligibility guidelines and any intended use of professional writers	Line 389-399	The section is written according to authorship eligibility guidelines.
Dissemination policy: reproducible research	#31c	Plans, if any, for granting public access to the full protocol, participant-level dataset, and statistical code	Line 387	The Study Protocol is currently only available in Swedish.
**Appendices**				

For peer review only

Informed consent materials	#32	Model consent form and other related documentation given to participants and authorised surrogates		Details not included in the manuscript but is available in the Study Protocol with the attached patient study information/consent form.
Biological specimens	#33	Plans for collection, laboratory evaluation, and storage of biological specimens for genetic or molecular analysis in the current trial and for future use in ancillary studies, if applicable	Line 214-263	

It is strongly recommended that this checklist be read in conjunction with the SPIRIT 2013 Explanation & Elaboration for important clarification on the items. Amendments to the protocol should be tracked and dated. The SPIRIT checklist is copyrighted by the SPIRIT Group under the Creative Commons "Attribution-NonCommercial-NoDerivs 3.0 Unported" license. This checklist can be completed online using https://www.goodreports.org/, a tool made by the EQUATOR Network in collaboration with Penelope.ai

For peer review only

BMJ Open

Safety and pharmacokinetics-pharmacodynamics of a shorter tuberculosis treatment with high-dose pyrazinamide and rifampicin – a study protocol of a phase II clinical trial (HighShort-RP)

Journal:	*BMJ Open*
Manuscript ID	bmjopen-2021-054788.R1
Article Type:	Protocol
Date Submitted by the Author:	23-Jan-2022
Complete List of Authors:	Ekqvist, David; Linköping University, Department of Infectious Diseases, Region Östergötland<br>Bornefall, Anna; Region Östergötland, Department of Infectious Diseases<br>Augustinsson, Daniel; Region Östergötland, Department of Infectious Diseases<br>Sönnerbrandt, Martina; Region Östergötland, Department of Infectious Diseases<br>Nordvall, Michaela Jonsson; Linköping University, Department of Clinical Microbiology, and Department of Biomedical and Clinical Sciences<br>Fredrikson, Mats; Linköping University, Forum Östergötland<br>Carlsson, Björn; Linköping University, Department of Clinical Pharmacology, and Department of Biomedical and Clinical Sciences<br>Sandstedt, Mårten; Linköping University, Department of Radiology in Linköping, and Department of Health, Medicine and Caring Sciences<br>Simonsson, Ulrika; Uppsala University, Department of Pharmaceutical Biosciences<br>Alffenaar, Jan-Willem; The University of Sydney Faculty of Medicine and Health, School of Pharmacy; The University of Sydney, Marie Bashir Institute of Infectious Diseases and Biosecurity<br>Paues, Jakob; Linköping University, Department of Infectious Diseases, and Department of Biomedical and Clinical Sciences<br>Niward, Katarina; Linköping University, Department of Infectious Diseases, and Department of Biomedical and Clinical Sciences
**Primary Subject Heading**:	Infectious diseases
Secondary Subject Heading:	Pharmacology and therapeutics
Keywords:	Tuberculosis < INFECTIOUS DISEASES, Clinical trials < THERAPEUTICS, Adverse events < THERAPEUTICS, CLINICAL PHARMACOLOGY

SCHOLARONE™
Manuscripts

Safety and pharmacokinetics-pharmacodynamics of a shorter tuberculosis treatment with high-dose pyrazinamide and rifampicin – a study protocol of a phase II clinical trial (HighShort-RP)

David Ekqvist[1], Anna Bornefall[1], Daniel Augustinsson[1], Martina Sönnerbrandt[1], Michaela Jonsson Nordvall[2], Mats Fredrikson[3], Björn Carlsson[4], Mårten Sandstedt[5], Ulrika S. H. Simonsson[6], Jan-Willem Alffenaar[7,8,9], Jakob Paues[10], Katarina Niward*[10]

1. Department of Infectious Diseases, Linköping University Hospital, Linköping, Sweden
2. Department of Clinical Microbiology, and Department of Biomedical and Clinical Sciences, Linköping University, Linköping, Sweden
3. Forum Östergötland, Linköping University, Linköping, Sweden
4. Department of Clinical Pharmacology, and Department of Biomedical and Clinical Sciences, Linköping University, Linköping, Sweden
5. Department of Radiology in Linköping, and Department of Health, Medicine and Caring Sciences, Linköping University, Linköping, Sweden
6. Department of Pharmaceutical Biosciences, Uppsala University, Sweden
7. University of Sydney, Faculty of Medicine and Health, School of Pharmacy, Sydney, Australia
8. Westmead hospital, Sydney, Australia
9. Marie Bashir Institute of Infectious Diseases and Biosecurity, University of Sydney, Sydney, Australia
10. Department of Infectious Diseases, and Department of Biomedical and Clinical Sciences, Linköping University, Linköping, Sweden

*Corresponding author: Dr Katarina Niward; katarina.niward@liu.se

Key words: pulmonary tuberculosis, pyrazinamide, rifampicin, pharmacokinetic-pharmacodynamics (PK-PD), adverse event (AE), time to culture positivity (TTP)

Word count: 3989

For peer review only

ABSTRACT

Introduction Increased dosing of rifampicin and pyrazinamide seems a viable strategy to shorten treatment and prevent relapse of drug-susceptible tuberculosis (TB), but safety and efficacy remains to be confirmed. This clinical trial aims to explore safety and pharmacokinetics-pharmacodynamics of a high-dose pyrazinamide-rifampicin regimen.

Methods and analysis Adult patients with pulmonary TB admitted to six hospitals in Sweden and subjected to receive first-line treatment, are included. Patients are randomised (1:3) to either 6-month standardised TB treatment or a 4-month regimen based on high-dose pyrazinamide (40 mg/kg) and rifampicin (35 mg/kg) along with standard doses of isoniazid and ethambutol. Plasma samples for measurement of drug exposure determined by liquid chromatography tandem-mass spectrometry are obtained at 0, 1, 2, 4, 6, 8, 12 and 24 h, at day 1 and 14. Maximal drug concentration (C_{max}) and area under the concentration-time curve (AUC_{0-24h}) are estimated by non-compartmental analysis. Conditions for early model-informed precision dosing of high-dose pyrazinamide-rifampicin are pharmacometrically explored. Adverse drug effects are monitored throughout the study and graded according to Common Terminology Criteria for Adverse Events v5.0. Early bactericidal activity is assessed by time to positivity in BACTEC MGIT 960 of induced sputum collected at day 0, 5, 8, 15 and week 8. Minimum inhibitory concentrations of first-line drugs are determined using broth microdilution. Disease severity is assessed with X-ray grading and a validated clinical scoring tool (TBscore II). Clinical outcome is registered according to WHO definitions (2020) in addition to occurrence of relapse after end of treatment. Primary endpoint is pyrazinamide AUC_{0-24h} and main secondary endpoint is safety.

For peer review only

Ethics and dissemination The study is approved by the Swedish Ethical Review Authority and the Swedish Medical Products Agency. Informed written consent is collected before study enrolment. The study results will be submitted to a peer-reviewed journal.

Trial registration number NCT04694586; Pre-results.

ARTICLE SUMMARY

Strengths and limitations of this study

- This phase II clinical trial of patients with drug-susceptible pulmonary tuberculosis (TB) will provide data on safety and early bactericidal activity in relation to drug exposure of an experimental shorter TB regimen based on high-dose pyrazinamide and rifampicin.
- The patients' drug exposure will be related to individual *Mycobacterium tuberculosis* minimal inhibitory concentrations (MICs), exploring pharmacokinetics-pharmacodynamics indices during high-dose pyrazinamide and rifampicin.
- Opportunities for early model-informed precision dosing after the first dose will be explored by pharmacokinetic modelling.
- A limitation of the study is the small sample size, due to an extensive and resource demanding study protocol, although this is in accordance with phase II studies and will partly be compensated for by using rich data.

For peer review only

INTRODUCTION

Current treatment of drug-susceptible tuberculosis (TB) is based on recommendations by the World Health Organization (WHO) and originates from the 1970s (1, 2). During the first two months (intensive phase) daily doses of isoniazid 5 mg/kg , rifampicin 10 mg/kg, pyrazinamide 25 mg/kg, and ethambutol 15 mg/kg are given, followed by a continuation phase of isoniazid and rifampicin (1). This 6-month multi-drug regimen is intended to prevent acquired drug resistance, therapy failure and relapse (3). Although effective, the long treatment duration increases the risk of non-compliance. Hence shorter and more effective regimens with preserved safety profile are needed as part of the global strategy to eliminate TB. Several attempts to shorten TB treatment below 6 months have been unsuccessful due to increased frequency of relapse (4-7). However, a pooled analysis of patient-level data from 4-month fluoroquinolone-containing regimens identified non-inferiority compared with standard treatment in participants with minimal non-cavitary disease (8). Recently, a 4-month rifapentine-based regimen containing moxifloxacin for treatment of pulmonary TB was non-inferior to standard 6-month TB regimen in terms of TB disease-free survival 12 months after randomisation (9).

Accumulating data from clinical trials exploring higher doses of rifampicin (10-13) have shown increased bactericidal activity with potential to shorten TB treatment corroborated by results from pharmacokinetic-pharmacodynamic (PK-PD) modelling and simulation (14, 15). Lately, pyrazinamide has attained increasing attention due to its potent sterilising activity with the ability to eliminate intracellular resting and slowly replicating *Mycobacterium tuberculosis* (*Mtb*) so-called "persisters" (16). Once the drug was added to standard TB treatment in the early 1980s the treatment length could be shortened from 9 to 6 months without increase in relapse (2, 17). Efforts to further shorten TB treatment from 6 to 4 months by simply prolonging the duration of pyrazinamide was unsuccessful because of increased frequency of relapse (18).

For peer review only

Observational studies of more than 100 patients with pulmonary TB (19, 20) and a recent meta-analysis (21) have shown that unsuccessful treatment outcome was associated with suboptimal pyrazinamide exposure. Pharmacokinetic studies in patients with pulmonary TB have shown lower than recommended drug concentration levels (22) with standard dosing of both rifampicin (42-80%) and pyrazinamide (27-53%) (23, 24). In a large PK-PD study evaluating patient-level data from three phase II clinical trials with pulmonary TB patients where the majority had cavitary disease, participants received rifampicin (range 10-35 mg/kg), pyrazinamide (range 20-30 mg/kg) and two companion drugs (25). The results showed that pyrazinamide's induction of culture negativity increased with augmented drug exposure. Hepatotoxicity was rare and not associated with pyrazinamide exposure. Furthermore, pharmacokinetic modelling suggested that a massive dose of pyrazinamide (>4500 mg) or increasing both pyrazinamide and rifampicin doses would be required to reach microbiological targets associated with treatment-shortening such as 90-95% culture conversion rate by 2 months of treatment (25). Rifampicin doses of at least three times the standard dose combined with isoniazid, pyrazinamide and ethambutol was safely administered to 63 patients with pulmonary TB for 12 weeks (11). Rifampicin 40 mg/kg was recently identified as the highest tolerated dose with dose limiting factors reported as gastrointestinal disorders, pruritis, hyperbilirubinemia and jaundice (26). However, the safety of simultaneously administered high-dose pyrazinamide and rifampicin remains to be explored. Our study aims to investigate PK-PD data, safety and tolerability of a 4-month first-line treatment with combined high dosing of pyrazinamide and rifampicin to a selected group of patients with non-advanced pulmonary TB.

METHODS AND ANALYSIS

Study design

For peer review only

We are conducting a multicentre open-label randomised controlled phase II study. The study protocol conforms with SPIRIT (Standard Protocol Items: Recommendations for Interventional Trials), an international format for interventional trials (27, 28).

Study setting

The study is carried out at infectious disease departments in six hospitals in Sweden, three tertiary level hospitals and three secondary level hospitals. The number of hospital beds in the hospitals range from 310 to 1340. The incidence of TB in Sweden is 4.75/100 000 (2019) and the vast majority (85%) of patients are migrants that have acquired their infection prior to arriving to Sweden (29). The number of new culture positive pulmonary TB cases during 2019 was 93 in total within the regions served by the six hospitals included in the study (29). A majority of the study participants will be admitted to a hospital ward as per standard of care for isolation of patients with smear positive pulmonary TB. Participants not considered in need of hospital admission or isolation will be followed by frequent outpatient visits as well as hospital admission on day 1 and 14 to facilitate the frequent PK-sampling. The study is registered at ClinicalTrials.gov (NCT04694586) and opened for recruitment 23rd of November 2020.

Study participants

A total number of 40 evaluable patients will be included. Study inclusion and exclusion criteria are listed in Table 1.

Table 1. Inclusion and exclusion criteria in HighShort-RP.

Inclusion criteria	*Exclusion criteria*
Adult (≥18 year)	Treated for TB within the last year.
Pulmonary TB confirmed by PCR and/or culture for *Mtb*.	Molecular or phenotypic resistance to any of the first-line oral drugs.
Eligible for first-line TB treatment, but not yet started treatment.	Miliary TB or advanced pulmonary TB with sputum smear grade 3 or advanced TB on chest X-ray according to National

For peer review only

For peer review only - http://bmjopen.bmj.com/site/about/guidelines.xhtml

	Tuberculosis Association, 1961, (30) defined by one or more of the following radiological findings:<br>- cavitation of >4 cm diameter<br>- disseminated lesions of slight to moderate density which extend throughout the total volume of more than one lung (uni- or bilateral)<br>- radiological signs of dense and/or confluent lesions affecting >1/3 of one lung or in both lungs combined
Hiv-negative	Any other infectious disease of such significance that urgent treatment is required.
BMI (Body Mass Index) >17	Ongoing treatment with other medication where treatment with rifampicin can interact with the medication and compensatory drug adjustments cannot be made.
Written informed consent to participate in the study.	Previously known allergy to any of the first-line drugs or other contraindications according to the Summary of Product Characteristics (SmPC) of rifampicin, isoniazid, pyrazinamide, and ethambutol.
Fertile women must agree to use a barrier contraceptive during the study and have a negative pregnancy test on inclusion.	Immunocompromised defined as primary or secondary immunodeficiency including immunosuppressive treatment such as chemotherapy, immunomodulating agents, corticosteroids equivalent to ≥15 mg prednisolone.
	Known liver disease or aspartate aminotransferase (AST) or alanine aminotransferase (ALT) >1.5 times the upper normal level (UNL).
	Heart failure (NYHA class III and IV)
	Kidney failure (eGFR <50 mL/min)
	Dysregulated diabetes mellitus
	Alcohol or drug abuse
	Pregnancy or breast feeding
	Bodyweight <35 or >90 kg

For peer review only

Study outline

The overall study outline is shown in Figure 1.

Figure 1. The study outline in the flow-chart arrow reflects study activity identical for the intervention and control group. After four months treatment, the intervention group is followed-up according to the follow-up information found in the bottom of the flow-chart arrow, meanwhile the control group is followed-up according to routine care (not included in the picture). Study participants are provided with a drug diary to record concomitant drugs, food intake and potential side-effects during the treatment. Sputum samples are collected regularly during the study to assess time to positivity (TTP) in BACTEC MGIT 960 for mycobacterial liquid culture. Rich blood sampling for measurement of drug concentrations is collected on the day of the first dose and after 14 days of treatment. Changes in clinical status is assessed by a clinical scoring tool (TBscore II) in addition to evaluation by chest X-ray and/or thoracic computed tomography. Safety data is monitored closely during the first four study months and is based on urgently reported serious adverse events, adverse event (AE) questionnaire, medical records, regularly surveillance of blood chemistry and during the first two weeks analysis of electrocardiogram (ECG). The final treatment outcome, according to WHO definitions and occurrence of relapse, is registered for both study arms after treatment completion.

After providing informed consent patients are screened for eligibility to participate in the study based on the inclusion and exclusion criteria (Table 1). A review and registration of the patient's clinical status and symptoms is made, and any pre-existing symptoms and findings are recorded for future reference as potential adverse event (AE) from high-dose pyrazinamide and rifampicin can overlap with the symptoms of active TB. Provided all the inclusion criteria and none of the exclusion criteria are fulfilled according to the responsible physician, the study participant is electronically randomised in a 1:3 ratio to either open-label standard first-line TB treatment for six months or to a shortened treatment of four months in the intervention group. The higher doses utilised in the intervention group are 40 mg/kg of pyrazinamide (for 8 weeks) and 35 mg/kg of rifampicin (for 4 months) and with standard dosing of isoniazid (5 mg/kg for 4 months) and ethambutol (15-20 mg/kg for 8 weeks) (Table 2). Dose adjustment based on changes in body weight will be evaluated after two weeks of therapy.

For peer review only

170 **Table 2.** Treatment in control and intervention group. Approved fixed dose combination tablets will be used 171 during the treatment period and in the intervention group combined with single tablets of pyrazinamide and 172 rifampicin to reach target doses of pyrazinamide 40 mg/kg (range 35-43 mg/kg) and rifampicin 35 mg/kg 173 (33-37 mg/kg) according to weight-based tables included in the study protocol.

Drug	*Control group*	*Intervention group*
Pyrazinamide	20-30 mg/kg/day for 8 weeks	40 mg/kg/day for 8 weeks
Rifampicin	10 mg/kg/day for 6 months	35 mg/kg/day for 4 months
Isoniazid	5 mg/kg/day for 6 months	5 mg/kg/day for 4 months
Ethambutol	15-20 mg/kg/day for 8 weeks	15-20 mg/kg/day for 8 weeks

174 Clinical evaluation of the participants and disease severity is assessed and documented 175 according to TBscore II (Table 3) as well as with chest X-ray (30, 31).

176 **Table 3.** Parameters used for the TBscore II (31).

Parameters	*Points assigned*
Self-reported	
Cough	1
Dyspnoea	1
Chest pain	1
Anaemia (pale conjunctiva)	1
BMI (Body Mass Index) <18	1
BMI <16	1
MUAC (Middle Upper Arm Circumference) <220 mm	1
MUAC <200 mm	1

177 First-line drug concentration measurements in plasma are determined at 0, 1, 2, 4, 6, 8, 12, and 178 24 hrs after drug intake on day 1 and day 14. Maximal drug concentration (C_{max}) and area under 179 the concentration-time curve ($AUC_{0\text{-}24h}$) are assessed by non-compartmental analysis using the 180 linear trapezoidal method (32). The potential for early model-informed precision dosing of

For peer review only

high-dose pyrazinamide and rifampicin will be explored by applying PK modelling on drug concentrations obtained after the first dose versus after 14 days of treatment. Induced sputum samples are collected on day 0, 5, 8, 15 and week 8.

Collection of safety measures and follow-up

All study participants are monitored throughout the study as per routine care. Additional study-specific visits and monitoring by electrocardiogram (ECG), blood sampling, sputum collection and radiology is outlined in Figure 1. Participants in the study will be provided with a diary to record drug intake and to note any suspected side-effects from the treatment. Safety data are collected during the intensive phase by weekly AE questionnaire interviews and from the patient diary, the medical record and by frequent laboratory monitoring for blood chemistry tests as displayed in Figure 1. During the continuation phase the same safety routine is applied every second week. Adverse events are graded based on Common Terminology Criteria for Adverse Events v 5.0 (CTCAE v 5.0). A Serious Adverse Event (SAE) needs to be reported within 24 hours after the study team has been notified and is defined as death of any cause, life-threatening AE and AE that results in prolonged or new hospital admission or permanent disability. For safety reasons, AE considered to be related to TB treatment and causes cessation of pyrazinamide and/or rifampicin, needs to be reported in the same manner as SAE. Based on these reports a decision is made if the SAE requires any further action in terms of trial continuation. Any new concomitant medication is recorded and evaluated to check for potential interactions with the TB treatment. As an additional safety measure, sputum samples collected during the intensive phase will be used to evaluate the effect of combined high-dose treatment on early bactericidal activity (EBA) by analysis of time to positivity (TTP) automatically detected in BACTEC MGIT 960 (MGIT) for mycobacterial liquid culture. Adequate microbiological response is defined as sputum culture conversion between day 0 and 15 or difference in TTP between day 0 and 5 by at least 25 hours, day 0 and 8 by 50 hours or day 0

For peer review only

and 15 by 75 hours based on unpublished data of TTP-dynamics observed in our observational cohort study (24) and the literature (33-35).

Study participants randomised to the intervention group will be followed for a total of 28 months to record any signs of relapse. Study participants randomised to standard of care will have a follow-up visit 6 to 12 months after completion of treatment and any signs of relapse beyond that point will be retrieved by data collection from the Swedish national tuberculosis registry 24 months after treatment completion. In addition to registration of relapse, final treatment outcome is recorded according to WHO definitions (36).

Bioanalytic methods

Drug concentration measurement

Blood is collected in 6 ml sodium-heparin blood tubes, centrifuged at 2000 g for 10 min within 1 hour from sampling. Aliquots of plasma is frozen in –70°C pending analysis, which will be made every three months (37, 38) at the Department of Clinical Pharmacology, Linköping University Hospital, Sweden (accredited ISO/IEC 17025:2018). Total drug concentration of isoniazid, acetyl isoniazid, rifampicin, pyrazinamide, and ethambutol will be determined by preparing samples with organic solvent protein precipitation in accordance with Fang et al and Han et al (39, 40). The samples will be analysed with liquid chromatography on a reversed phase column ACQUITY UPLC BEH C18 Column, 130Å, 1.7 µm, 2.1 mm X 50 mm a BEH C18 VanGuard pre-column and column in-line Filter (Waters Corporation, Milford, USA) and with tandem-mass spectrometry MS detection using a tandem quadrupole mass spectrometer (Xevo TQ MS, Waters) in positive electrospray ionization mode with the following quantification traces; isoniazid 137.96 > 121.04, acetyl isoniazid 180.0 > 121.0, rifampicin 823.30 > 791.416, pyrazinamide 123.95 > 54.1, and ethambutol 205.1 > 116.15. The analytic range for isoniazid is 0.25-8 mg/l, acetyl isoniazid 0.25-8 mg/l, rifampicin 2.5-80 mg/l,

For peer review only

230 pyrazinamide 2.5-80 mg/l, and ethambutol 0.25-8 mg/l. The drug concentration analysis
231 method is validated according to EMA guideline on bioanalytical method validation (41). For
232 the quality controls at 0.75 mg/l and 6 mg/l for isoniazid, acetyl isoniazid and ethambutol the
233 inter-day accuracy is between 95.8-99.9 % and with a precision between 7.7-10.5 % and for
234 pyrazinamide and rifampicin quality controls at 7.5 and 60 mg/l the accuracy is between 92.3-
235 97.6 % and precision between 9.3-11.8 %. Intra-day at 0.75 mg/l and 6 mg/l for isoniazid, acetyl
236 isoniazid and ethambutol gave accuracy between 86.7-95.6 % and a precision of 0.6-5.2 % and
237 for pyrazinamide and rifampicin at 7.5 and 60 mg/l an accuracy between 92.3-108.8 % and
238 precision of 2.2-7.5 %.

239 **Microbiology**

240 *Time to positivity (TTP)*

241 Sputum samples are treated according to routine and are carried out at a biosafety level 3
242 laboratory at the accredited TB laboratory of the Department of Clinical Microbiology at
243 Linköping University Hospital, Sweden. In short, following treatment of sputum with
244 sputolysine, samples are centrifuged and resuspended in 1.5% NaOH-SDS with 2 mm glass
245 beads and vigorously stirred using a vortex mixer. After neutralisation by 0.09% sulfuric acid
246 samples are washed in phosphate buffer and added to MGIT-tubes according to the instructions
247 by the manufacturer. The MGIT-tubes are incubated using BACTEC MGIT 960 (Becton
248 Dickinson, Lakes, NJ, USA) at 37°C for up to 42 days. When growth is detected TTP is
249 automatically recorded, *Mtb* isolates are stored at -70°C awaiting MIC determination.

250 *Minimum inhibitory concentrations (MIC)*

251 Previously stored *Mtb* isolates (-70°C) from study participant obtained before treatment
252 initiation will be used for MIC determination of first-line drugs. MIC determination will be
253 performed for rifampicin, isoniazid and ethambutol by broth microdilution in accordance with

For peer review only

the EUCAST reference method for *Mtb*, and when applicable additional group A drugs as displayed in Figure 2 (42). MIC is defined as the lowest antibiotic concentration without growth when the 1:100 diluted controls show visual growth. *M. tuberculosis* H37Rv ATCC 27294 will be included as an internal quality control in each batch. Breakpoints established by the Clinical and Laboratory Standards Institute (CSLI) are used for drug susceptibility testing (DST) of isolates for rifampicin, isoniazid, and ethambutol in broth microdilution plates (1, 0.12 and 8 mg/L, respectively) (43). Routine DST of first-line drugs and MIC determination of pyrazinamide are performed in MGIT as previously described with WHO-based critical concentrations of antibiotics for routine DST (rifampicin 0.5, isoniazid 0.1, pyrazinamide 100, and ethambutol 5 mg/L) and by using serial 2-fold dilutions of antibiotics for pyrazinamide MIC testing (44). Pyrazinamide will also be included in the reference method (pH 5.9) as an internal validation (Figure 2).

Figure 2. Plate outline for MIC determination by broth microdilution using the EUCAST reference method. dH_2O=distilled water, GC=growth control, RIF=rifampicin, INH=isoniazid, LEV=levofloxacin, PZA=pyrazinamide, EMB=ethambutol, LIN=linezolid

Data analysis plan

Study monitoring is performed regularly by the regional support unit for clinical trials, Forum Östergötland, at all study sites before study opening, during recruitment period and at study closure. Obtained study data are entered in electronic case record forms and managed by using REDCap electronic data capture tools (45, 46) hosted at Linköping University and at time of data analysis transferred to a database for statistical processing.

The primary outcome is $AUC_{0\text{-}24h}$ of pyrazinamide in the intervention arm and the main secondary outcome is the incidence and severity of adverse events. The distribution of $AUC_{0\text{-}24h}$, C_{max} and PK-PD indices ($AUC_{0\text{-}24h}$/MIC, C_{max}/MIC) will be visualised separately for the two study arms. Difference in drug exposure will be explored by Mann-Whitney's U-test. A p-

For peer review only

value of ≤0.05 will be considered statistically significant. The PK-PD indices based on drug exposure at day 14 for the investigational products (pyrazinamide and rifampicin) will be correlated to tentative PK-PD targets derived from the literature on human studies such as the suggested $AUC_{0\text{-}24h}$ >363 mg*h/L and AUC/MIC >8.42 for pyrazinamide (47). Associations between drug exposure and liver laboratory assessments will be analysed by using Spearman rank correlation. The incidence of minimum grade two AE and SAE will be presented in frequency tables in addition to correlation graphs on exposure-tolerability. Nonlinear mixed effects modelling will be utilised to derive a population PK model for high-dose pyrazinamide. A model-informed precision dosing approach will be derived in order to be able to predict optimised dose given information from early PK sampling. An earlier developed model-informed precision dosing approach for high-dose rifampicin (48, 49) will be applied to evaluate the accuracy in the high-dose predictions. The sample size of 10 participants in the control group and 30 in the intervention group will enable sufficient descriptive PK data of the combined high-dose pyrazinamide and rifampicin regimen accounting for the expected larger PK-variability in this group compared with the control group (49). Applying EBA as one of the safety endpoints, we estimate 30% difference in EBA between the study arms (80% power, p=0.05), based on previous unpublished data of TTP-dynamics observed in our observational cohort study (24) combined with EBA data from the literature (10, 11, 50). Randomisation is performed in blocks stratified by gender and will continue until 10 individuals in the control arm and 30 individuals in the experimental arm have completed PK sampling day 14. The randomisation scheme was done with the statistical program R version 4 (the R foundation) by an independent statistician, uploaded into the CRF-program REDCap (45, 46) and blinded to the person who includes patients. Resistance to first-line drugs is rare in Sweden and multidrug resistant (MDR) TB accounted for 1.8% of all new TB cases 2019 (29) and the majority of the drug resistant cases are detected early. Thus, late exclusions from the study based on detected

For peer review only

drug resistance, are expected to be negligible. A modified -to-treat analysis will be performed for patients who may miss follow-up in addition to a per-protocol analysis.

Patient and public involvement

Patients were not involved in the development of the research plan nor the implementation of this clinical trial. We aim to present study results at a conference where patient representatives or patient communities are present. Furthermore, patients will be involved in a future phase III trial based on the results of this study.

DISCUSSION

There is strong urge for a shortened treatment of drug-susceptible TB. In this phase II clinical trial (HighShort-RP) of a shorter experimental TB regimen based on high-dose pyrazinamide and rifampicin, tolerability and safety will be investigated along with in-depth PK-PD analysis including determination of individual MICs. In a PK-PD modelling study published just after our study opened for recruitment, the relationship between pyrazinamide exposures and efficacy or hepatotoxicity was assessed on pooled retrospective data from three clinical interventional trials conducted by the Tuberculosis Trials Consortium and Pan African Consortium for the Evaluation of Antituberculosis Antibiotics Networks (25). Simulations identified two potentially treatment-shortening strategies restricted to currently used first-line drugs whereof one was to optimise drug exposure of rifampicin and pyrazinamide in parallel. The authors stated that whether or not an enhanced multidrug regimen based on high-dose rifampicin (e.g. ≥35 mg/kg) and higher-dose pyrazinamide (e.g. 30-40 mg/kg) will be sufficient to significantly reduce the length of TB treatment must be explored prospectively, with attention to safety and tolerability. Results from our clinical trial will be indicative whether the combined high-dose rifampicin (35 mg/kg) with pyrazinamide (40 mg/kg) along with standard doses of isoniazid and ethambutol, is tolerable for patients. Furthermore, the PK-PD data retrieved from

For peer review only

329 the study can be included in predictions of effective treatment-shortening regimens launched in
330 larger RCTs evaluating high-dose sterilising regimens with non-inferiority criteria compared
331 with standard of care. Recently, a large RCT of pulmonary TB patients showed that the efficacy
332 of a 4-month rifapentine-based regimen containing moxifloxacin was noninferior to standard
333 6-month TB regimen in terms of TB disease-free survival 12 months after randomisation (9).
334 Our long-term goal is to achieve maximum output of the first-line drugs and if possible, to avoid
335 a fluoroquinolone-based treatment-shortening regimen.

336 The sample size of this phase II study is small as the study protocol is extensive and resource
337 demanding. Each participant is closely monitored for safety in addition to frequent PK sampling
338 during the first 14 days of the study allowing a detailed description of PK parameters. The
339 clinical scoring system TBscore II used in the study to grade disease severity and assess
340 treatment response was originally developed and validated for use in low-income settings and
341 therefore scoring is based on clinical signs rather than laboratory results (31).

342 Personalised medicine based on disease severity and dose-adjustments guided by therapeutic
343 drug monitoring to achieve individual maximum exposures of TB drugs is promising, in
344 particularly as the variation of drug exposures is extensive during high-dose rifampicin
345 treatment (10, 15). This highlights the value of measuring drug exposure and the prerequisites
346 for early model-informed precision dosing based on PK data obtained after the first dose will
347 be explored in this phase II clinical trial.

348 **Ethics and dissemination**

349 This study has been approved by the Swedish Ethical Review Authority (approval number
350 2020-00761) and the Swedish Medical Products Agency (approval number 5.1-2019-105207
351 and EudraCT number 2019-003721-25). Prior to the study opening, a designated study team of
352 nurses, physiotherapists (training of study nurses in collection of induced sputum), laboratory

For peer review only

staff and clinical doctors participated in training sessions of the study protocol which included study-specific procedures and ethical considerations. Study initiation meeting and the main training sessions were led by the coordinating principal investigator assisted by the support unit for clinical trials Forum Östergötland. Physicians and nurses in the study team have documented training in Good Clinical Practice (GCP) and the study is performed in accordance with GCP and the Declaration of Helsinki. Informed consent is obtained from each study participant by a physician prior to any study-specific activity. Inclusion of patients in the study is restricted to the available set of written informed consent translated into selected languages. In addition and when required, an interpreter will be used for providing study information and to translate during study visits. Study participants are free to withdraw from the study at any time and will at that point be offered treatment according to standard of care. The safety of combined high-dose pyrazinamide (40 mg/kg) and rifampicin (35 mg/kg) has not been studied in humans before. However, TB treatment with rifampicin 35 mg/kg given to 63 patients for 12 weeks has been proven safe (11). Furthermore, data was recently presented on exposure-tolerability of two weeks treatment with rifampicin 40 mg/kg (n=15) and 50 mg/kg (n=17) where the dose of 40 mg/kg was safe and tolerable (26). In early studies on lengthy treatments of pyrazinamide beyond 8 weeks, increased liver markers, jaundice and arthralgia were associated with higher doses of pyrazinamide (51). In a meta-analysis comparing rates of AEs between different doses of pyrazinamide, the frequencies of hepatotoxicity were 0.042 (95% CI, 0.026 to 0.067) for 30 mg/kg, 0.055 (95% CI, 0.031 to 0.094) for 40 mg/kg, and 0.098 (95% CI, 0.047 to 0.193) for 60 mg/kg, suggesting a considerable proportion of hepatoxicity rates may be idiosyncratic rather than dose-dependent (52). According to a meta-analysis (n=4490), liver toxicity did not seem to be dose-dependent until pyrazinamide exposures exceeded a weekly AUC of 5000 mg*h/L (53) compared to an AUC of 363 mg*h/L on standard dosing (47). Recently, a patient-level data analysis where patients received rifampicin (range 10-35 mg/kg), pyrazinamide

For peer review only

(range 20-30 mg/kg), plus two companion drugs, found liver toxicity to be rare (3.9% with Grade 3 or higher liver function tests, LFT), and no relationship was observed between pyrazinamide C_{max} and LFT levels (25). Furthermore, currently used doses of 30-40 mg/kg pyrazinamide daily as part of MDR-TB treatment are well-tolerated (54). In summary, we consider it safe for patients to participate in our clinical trial and study participants will be closely monitored regarding potential side effects including weekly measurement of LFT during the first 8 weeks when high-dose pyrazinamide and rifampicin are combined. As this is a phase II clinical trial exploring increased dosing of already approved and widely used drugs, the review authorities did not require a data safety and monitoring board (DMSB).

Regardless of study results, we plan to present our data in international conferences and to publish our results in a peer-reviewed journal. Any significant protocol amendments will be reported to the ethical review authority and medical products agency.

Data sharing statement

No data are currently available.

Author's contribution

The study design was developed within the TB research group at Linköping University Hospital led by JP and KN with support from JWA and USHS on the pharmacokinetic part of the study. Statistician MF assisted in statistical selection of study dimensions and developed the block randomisation procedure. AB, JP and KN wrote the study protocol and the applications to ethical review authority and medical product agency. KN is the coordinating principal investigator of the clinical trial and research nurse in lead is MS. KN developed case record forms (CRFs), standard operating procedures (SOPs), led study initiation meeting with training sessions together with DE and assisted by DA, MS, MJN, MSa and JP. BC is the contact person at the laboratory of the Department of Clinical Pharmacology and MJN at the TB laboratory of

For peer review only

the Department of Clinical Microbiology. DE and KN wrote the first draft of the manuscript. All authors revised and approved the final version of the manuscript.

Funding statements

This work was supported by the Swedish National Research Council (KN, grant number 2019-05912), the Swedish Heart Lung Foundation (JP, grant number 20200302), the Research Council of south-eastern Sweden (JP, grant number FORSS-931028) and the Region Östergötland ALF grant (JP, grant number RÖ-888331). The funders had no role in any of the stages of the study.

Competing interest

None to declare.

Acknowledgments

We thank all members of the study team at the Infectious Diseases Clinic of Östergötland, former research nurse Lisa Gunnarsson at the Department of Infectious Diseases at Linköping University Hospital, initiated local principal investigators and the research coordinator Lina Malm at Forum Östergötland, Linköping University.

For peer review only

REFERENCES

1. World Health Organization. Guidelines for treatment of drug-susceptible tuberculosis and patient care (2017 update). Available from: https://www.who.int/tb/publications/2017/dstb_guidance_2017/en/.
2. Controlled clinical trial of short-course (6-month) regimens of chemotherapy for treatment of pulmonary tuberculosis. Lancet. 1972;1(7760):1079-85.
3. Fox W, Ellard GA, Mitchison DA. Studies on the treatment of tuberculosis undertaken by the British Medical Research Council tuberculosis units, 1946-1986, with relevant subsequent publications. Int J Tuberc Lung Dis. 1999;3(10 Suppl 2):S231-79.
4. Merle CS, Fielding K, Sow OB, Gninafon M, Lo MB, Mthiyane T, et al. A four-month gatifloxacin-containing regimen for treating tuberculosis. N Engl J Med. 2014;371(17):1588-98.
5. Gillespie SH, Crook AM, McHugh TD, Mendel CM, Meredith SK, Murray SR, et al. Four-month moxifloxacin-based regimens for drug-sensitive tuberculosis. N Engl J Med. 2014;371(17):1577-87.
6. Jawahar MS, Banurekha VV, Paramasivan CN, Rahman F, Ramachandran R, Venkatesan P, et al. Randomized clinical trial of thrice-weekly 4-month moxifloxacin or gatifloxacin containing regimens in the treatment of new sputum positive pulmonary tuberculosis patients. PLoS One. 2013;8(7):e67030.
7. Jindani A, Harrison TS, Nunn AJ, Phillips PP, Churchyard GJ, Charalambous S, et al. High-dose rifapentine with moxifloxacin for pulmonary tuberculosis. N Engl J Med. 2014;371(17):1599-608.
8. Imperial MZ, Nahid P, Phillips PPJ, Davies GR, Fielding K, Hanna D, et al. A patient-level pooled analysis of treatment-shortening regimens for drug-susceptible pulmonary tuberculosis. Nat Med. 2018;24(11):1708-15.
9. Dorman SE, Nahid P, Kurbatova EV, Phillips PPJ, Bryant K, Dooley KE, et al. Four-Month Rifapentine Regimens with or without Moxifloxacin for Tuberculosis. New England Journal of Medicine. 2021;384(18):1705-18.
10. Boeree MJ, Diacon AH, Dawson R, Narunsky K, du Bois J, Venter A, et al. A dose-ranging trial to optimize the dose of rifampin in the treatment of tuberculosis. Am J Respir Crit Care Med. 2015;191(9):1058-65.
11. Boeree MJ, Heinrich N, Aarnoutse R, Diacon AH, Dawson R, Rehal S, et al. High-dose rifampicin, moxifloxacin, and SQ109 for treating tuberculosis: a multi-arm, multi-stage randomised controlled trial. Lancet Infect Dis. 2017;17(1):39-49.
12. Aarnoutse RE, Kibiki GS, Reither K, Semvua HH, Haraka F, Mtabho CM, et al. Pharmacokinetics, Tolerability, and Bacteriological Response of Rifampin Administered at 600, 900, and 1,200 Milligrams Daily in Patients with Pulmonary Tuberculosis. Antimicrob Agents Chemother. 2017;61(11).
13. Velasquez GE, Brooks MB, Coit JM, Pertinez H, Vargas Vasquez D, Sanchez Garavito E, et al. Efficacy and Safety of High-Dose Rifampin in Pulmonary Tuberculosis. A Randomized Controlled Trial. Am J Respir Crit Care Med. 2018;198(5):657-66.
14. Svensson EM, Svensson RJ, Te Brake LHM, Boeree MJ, Heinrich N, Konsten S, et al. The Potential for Treatment Shortening With Higher Rifampicin Doses: Relating Drug Exposure to Treatment Response in Patients With Pulmonary Tuberculosis. Clin Infect Dis. 2018;67(1):34-41.
15. Svensson RJ, Svensson EM, Aarnoutse RE, Diacon AH, Dawson R, Gillespie SH, et al. Greater Early Bactericidal Activity at Higher Rifampicin Doses Revealed by Modeling and Clinical Trial Simulations. J Infect Dis. 2018;218(6):991-9.
16. Zhang Y, Shi W, Zhang W, Mitchison D. Mechanisms of Pyrazinamide Action and Resistance. Microbiol Spectr. 2014;2(4):Mgm2-0023-2013.

For peer review only

17. Controlled trial of 6-month and 8-month regimens in the treatment of pulmonary tuberculosis. First report. Am Rev Respir Dis. 1978;118(2):219-28.

18. Clinical trial of six-month and four-month regimens of chemotherapy in the treatment of pulmonary tuberculosis: the results up to 30 months. Tubercle. 1981;62(2):95-102.

19. Chideya S, Winston CA, Peloquin CA, Bradford WZ, Hopewell PC, Wells CD, et al. Isoniazid, rifampin, ethambutol, and pyrazinamide pharmacokinetics and treatment outcomes among a predominantly HIV-infected cohort of adults with tuberculosis from Botswana. Clin Infect Dis. 2009;48(12):1685-94.

20. Pasipanodya JG, McIlleron H, Burger A, Wash PA, Smith P, Gumbo T. Serum drug concentrations predictive of pulmonary tuberculosis outcomes. J Infect Dis. 2013;208(9):1464-73.

21. Perumal R, Naidoo K, Naidoo A, Ramachandran G, Requena-Mendez A, Sekaggya-Wiltshire C, et al. A systematic review and meta-analysis of first-line tuberculosis drug concentrations and treatment outcomes. Int J Tuberc Lung Dis. 2020;24(1):48-64.

22. Alsultan A, Peloquin CA. Therapeutic drug monitoring in the treatment of tuberculosis: an update. Drugs. 2014;74(8):839-54.

23. Rockwood N, Pasipanodya JG, Denti P, Sirgel F, Lesosky M, Gumbo T, et al. Concentration-Dependent Antagonism and Culture Conversion in Pulmonary Tuberculosis. Clin Infect Dis. 2017;64(10):1350-9.

24. Niward K, Davies Forsman L, Bruchfeld J, Chryssanthou E, Carlstrom O, Alomari T, et al. Distribution of plasma concentrations of first-line anti-TB drugs and individual MICs: a prospective cohort study in a low endemic setting. J Antimicrob Chemother. 2018;73(10):2838-45.

25. Zhang N, Savic RM, Boeree MJ, Peloquin C, Weiner M, Heinrich N, et al. Optimising pyrazinamide for the treatment of tuberculosis. Eur Respir J. 2021.

26. Te Brake LHM, De Jager V, Narunsky K, Vanker N, Svensson EM, Phillips PPJ, et al. Increased bactericidal activity but dose-limiting intolerability at 50 mg·kg−1 rifampicin. European Respiratory Journal. 2021:2000955.

27. Chan AW, Tetzlaff JM, Altman DG, Laupacis A, Gøtzsche PC, Krleža-Jerić K, et al. SPIRIT 2013 statement: defining standard protocol items for clinical trials. Ann Intern Med. 2013;158(3):200-7.

28. Chan AW, Tetzlaff JM, Gøtzsche PC, Altman DG, Mann H, Berlin JA, et al. SPIRIT 2013 explanation and elaboration: guidance for protocols of clinical trials. Bmj. 2013;346:e7586.

29. Folkhälsomyndigheten. Tuberkulos 2019 – utökad version. Available from: https://www.folkhalsomyndigheten.se/folkhalsorapportering-statistik/statistik-a-o/sjukdomsstatistik/tuberkulos/arsrapporter-och-kommentarer/2019/2019/ Accessed 2021-05-13.

30. American Thoracic Society. National Tuberculosis Association of the USA. Diagnostic Standards and Classification of Tuberculosis New York, NY, USA: National Tuberculosis Association; 1961.

31. Rudolf F, Lemvik G, Abate E, Verkuilen J, Schon T, Gomes VF, et al. TBscore II: refining and validating a simple clinical score for treatment monitoring of patients with pulmonary tuberculosis. Scand J Infect Dis. 2013;45(11):825-36.

32. Rowland M, Tozer TN. Clinical pharmacokinetics and pharmacodynamics; Concepts and applications, 4th ed. Philadelphia USA: Lippincott Williams and Wilkins; 2011.

33. Diacon AH, Dawson R, von Groote-Bidlingmaier F, Symons G, Venter A, Donald PR, et al. 14-day bactericidal activity of PA-824, bedaquiline, pyrazinamide, and moxifloxacin combinations: a randomised trial. The Lancet. 2012;380(9846):986-93.

34. Diacon A, Miyahara S, Dawson R, Sun X, Hogg E, Donahue K, et al. Assessing whether isoniazid is essential during the first 14 days of tuberculosis therapy: a phase 2a, open-label, randomised controlled trial. The Lancet Microbe. 2020;1(2):e84-e92.

For peer review only

35. Sabiiti W, Azam K, Farmer ECW, Kuchaka D, Mtafya B, Bowness R, et al. Tuberculosis bacillary load, an early marker of disease severity: the utility of tuberculosis Molecular Bacterial Load Assay. Thorax. 2020;75(7):606-8.
36. World Health Organization. Definitions and reporting framework for tuberculosis 2013 revision, updated December 2014 and January 2020, Geneva: World Health Organization Available from: https://www.who.int/publications/i/item/9789241505345.
37. Le Guellec C, Gaudet ML, Lamanetre S, Breteau M. Stability of rifampin in plasma: consequences for therapeutic monitoring and pharmacokinetic studies. Ther Drug Monit. 1997;19(6):669-74.
38. Zhou Z, Wu X, Wei Q, Liu Y, Liu P, Ma A, et al. Development and validation of a hydrophilic interaction liquid chromatography–tandem mass spectrometry method for the simultaneous determination of five first-line antituberculosis drugs in plasma. Analytical and Bioanalytical Chemistry. 2013;405(19):6323-35.
39. Fang P-F, Cai H-L, Li H-D, Zhu R-H, Tan Q-Y, Gao W, et al. Simultaneous determination of isoniazid, rifampicin, levofloxacin in mouse tissues and plasma by high performance liquid chromatography–tandem mass spectrometry. Journal of Chromatography B. 2010;878(24):2286-91.
40. Han M, Jun SH, Lee JH, Park KU, Song J, Song SH. Method for simultaneous analysis of nine second-line anti-tuberculosis drugs using UPLC-MS/MS. Journal of Antimicrobial Chemotherapy. 2013;68(9):2066-73.
41. European Medicines Agency. Guideline on bioanalytical method validation, 21 July 2011, 192217.
42. Schön T, Werngren J, Machado D, Borroni E, Wijkander M, Lina G, et al. Antimicrobial susceptibility testing of Mycobacterium tuberculosis complex isolates - the EUCAST broth microdilution reference method for MIC determination. Clin Microbiol Infect. 2020;26(11):1488-92.
43. CLSI. Performance Standards for Susceptibility Testing of Mycobacteria, Nocardia spp., and Other Aerobic Actinomycetes. 1st ed. CLSI supplement. Wayne, PA: Clinical and Laboratory Standards Institute; 2018.
44. Sturegard E, Angeby KA, Werngren J, Jureen P, Kronvall G, Giske CG, et al. Little difference between minimum inhibitory concentrations of Mycobacterium tuberculosis wild-type organisms determined with BACTEC MGIT 960 and Middlebrook 7H10. Clin Microbiol Infect. 2015;21(2):148.e5-7.
45. Harris PA, Taylor R, Thielke R, Payne J, Gonzalez N, Conde JG. Research electronic data capture (REDCap)—A metadata-driven methodology and workflow process for providing translational research informatics support. Journal of Biomedical Informatics. 2009;42(2):377-81.
46. Harris PA, Taylor R, Minor BL, Elliott V, Fernandez M, O'Neal L, et al. The REDCap consortium: Building an international community of software platform partners. Journal of Biomedical Informatics. 2019;95:103208.
47. Sturkenboom MGG, Märtson AG, Svensson EM, Sloan DJ, Dooley KE, van den Elsen SHJ, et al. Population Pharmacokinetics and Bayesian Dose Adjustment to Advance TDM of Anti-TB Drugs. Clin Pharmacokinet. 2021:1-26.
48. Svensson RJ, Niward K, Davies Forsman L, Bruchfeld J, Paues J, Eliasson E, et al. Individualised dosing algorithm and personalised treatment of high-dose rifampicin for tuberculosis. Br J Clin Pharmacol. 2019;85(10):2341-50.
49. Svensson RJ, Aarnoutse RE, Diacon AH, Dawson R, Gillespie SH, Boeree MJ, et al. A Population Pharmacokinetic Model Incorporating Saturable Pharmacokinetics and Autoinduction for High Rifampicin Doses. Clin Pharmacol Ther. 2018;103(4):674-83.
50. Jindani A, Dore CJ, Mitchison DA. Bactericidal and sterilizing activities of antituberculosis drugs during the first 14 days. Am J Respir Crit Care Med. 2003;167(10):1348-54.

For peer review only

51. Girling DJ. The hepatic toxicity of antituberculosis regimens containing isoniazid, rifampicin and pyrazinamide. Tubercle. 1978;59(1):13-32.
52. Pasipanodya JG, Gumbo T. Clinical and toxicodynamic evidence that high-dose pyrazinamide is not more hepatotoxic than the low doses currently used. Antimicrob Agents Chemother. 2010;54(7):2847-54.
53. Sahota T, Della Pasqua O. Feasibility of a fixed-dose regimen of pyrazinamide and its impact on systemic drug exposure and liver safety in patients with tuberculosis. Antimicrob Agents Chemother. 2012;56(11):5442-9.
54. Borisov S, Danila E, Maryandyshev A, Dalcolmo M, Miliauskas S, Kuksa L, et al. Surveillance of adverse events in the treatment of drug-resistant tuberculosis: first global report. Eur Respir J. 2019;54(6).

For peer review only

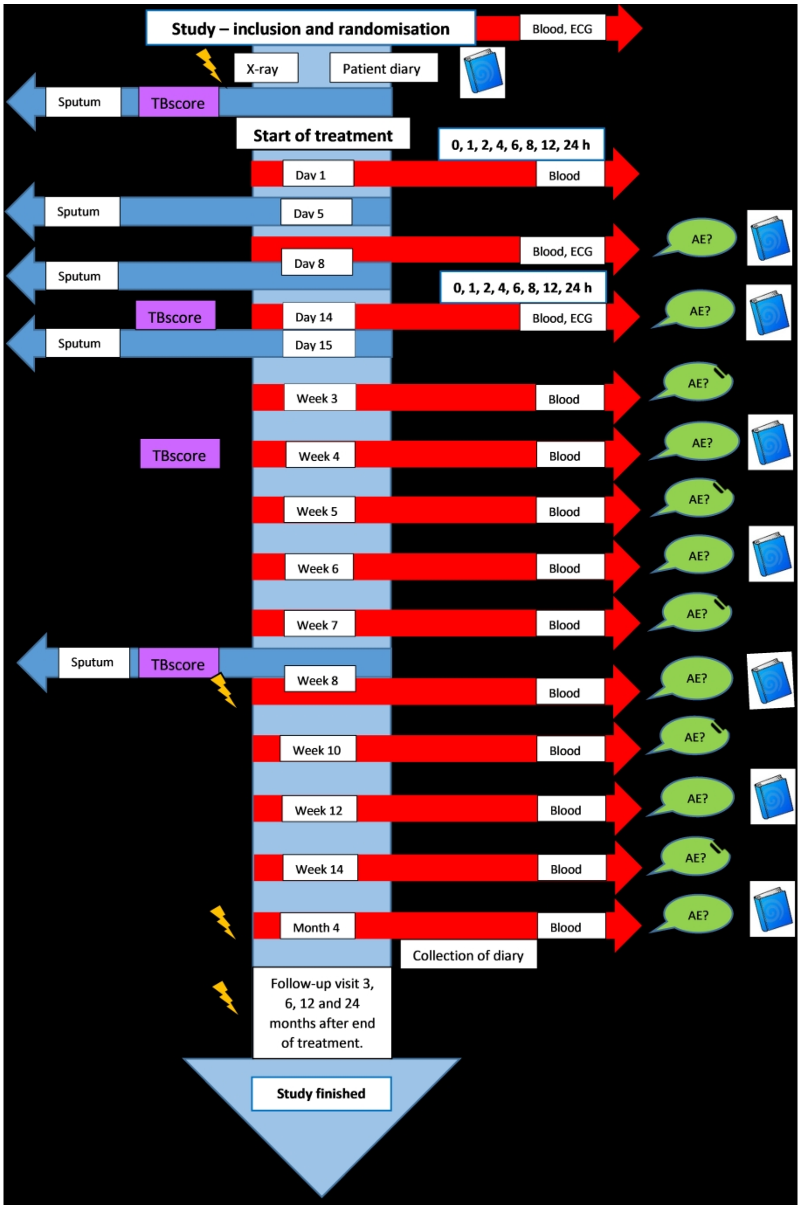

Figure 1. The study outline in the flow-chart arrow reflects study activity identical for the intervention and control group. After four months treatment, the intervention group is followed-up according to the follow-up information found in the bottom of the flow-chart arrow, meanwhile the control group is followed-up according to routine care (not included in the picture). Study participants are provided with a drug diary to record concomitant drugs, food intake and potential side-effects during the treatment. Sputum samples are collected regularly during the study to assess time to positivity (TTP) in BACTEC MGIT 960 for mycobacterial liquid culture. Rich blood sampling for measurement of drug concentrations is collected on the day of the first dose and after 14 days of treatment. Changes in clinical status is assessed by a clinical scoring tool (TBscore II) in addition to evaluation by chest X-ray and/or thoracic computed tomography. Safety data is monitored closely during the first four study months and is based on urgently reported serious adverse events, adverse event (AE) questionnaire, medical records, regularly surveillance of blood chemistry and during the first two weeks analysis of electrocardiogram (ECG). The final treatment outcome, according to WHO definitions and occurrence of relapse, is registered for both study arms after treatment completion.

159x240mm (600 x 600 DPI)

	1	2	3	4	5	6	7	8	9	10	11	12
A	200ul dH$_2$O	200ul dH$_2$O	200ul dH$_2$O	200ul dH$_2$O	200ul dH$_2$O	200ul dH$_2$O	200ul dH$_2$O	200ul dH$_2$O	200ul dH$_2$O	200ul dH$_2$O	200ul dH$_2$O	200ul dH$_2$O
B	negative control	GC 100%	RIF 0.032	RIF 0.064	RIF 0.125	RIF 0.25	RIF 0.5	RIF 1	RIF 2	RIF 4	GC 0.1%	200ul dH$_2$O
C	negative control	GC 100%	INH 0.016	INH 0.032	INH 0.06	INH 0.125	INH 0.25	INH 0.5	INH 1	INH 2	GC 1%	200ul dH$_2$O
D	negative control	GC 100%	LEV 0,03	LEV 0,06	LEV 0,12	LEV 0,25	LEV 0,5	LEV 1	LEV 2	LEV 4	GC 1%	200ul dH$_2$O
E	negative control	GC 1%	PZA 2	PZA 4	PZA 8	PZA 16	PZA 32	PZA 64	PZA 128	PZA 256	GC 10%	200ul dH$_2$O
F	negative control	GC 1%	EMB 0.125	EMB 0.25	EMB 0.5	EMB 1	EMB 2	EMB 4	EMB 8	EMB 16	GC 100%	200ul dH$_2$O
G	negative control	GC 1%	LIN 0.064	LIN 0.125	LIN 0.25	LIN 0.5	LIN 1	LIN 2	LIN 4	LIN 8	GC 100%	200ul dH$_2$O
H	200ul dH$_2$O	200ul dH$_2$O	200ul dH$_2$O	200ul dH$_2$O	200ul dH$_2$O	200ul dH$_2$O	200ul dH$_2$O	200ul dH$_2$O	200ul dH$_2$O	200ul dH$_2$O	200ul dH$_2$O	200ul dH$_2$O

Figure 2. Plate outline for MIC determination by broth microdilution using the EUCAST reference method. dH2O=distilled water, GC=growth control, RIF=rifampicin, INH=isoniazid, LEV=levofloxacin, PZA=pyrazinamide, EMB=ethambutol, LIN=linezolid

630x354mm (236 x 236 DPI)

SPIRIT Checklist for *Trials*

Complete this checklist by entering the page and line numbers where each of the items listed below can be found in your manuscript.

Your manuscript may not currently address all the items on the checklist. Please modify your text to include the missing information. If you are certain that an item does not apply, please state "n/a" and provide a short explanation. **Leaving an item blank or stating "n/a" without an explanation will lead to your manuscript being returned before review.**

Upload your completed checklist as an additional file when you submit to *Trials*. You must reference this additional file in the main text of your protocol submission. The completed SPIRIT figure must be included within the main body of the protocol text and can be downloaded here: http://www.spirit-statement.org/schedule-of-enrolment-interventions-and-assessments/

In your methods section, please state that you used the SPIRIT reporting guidelines, and cite them as:

Chan A-W, Tetzlaff JM, Gøtzsche PC, Altman DG, Mann H, Berlin J, Dickersin K, Hróbjartsson A, Schulz KF, Parulekar WR, Krleža-Jerić K, Laupacis A, Moher D. SPIRIT 2013 Explanation and Elaboration: Guidance for protocols of clinical trials. BMJ. 2013;346:e7586

		Reporting Item	Page and Line Number	Reason if not applicable
**Administrative information**				
Title	#1	Descriptive title identifying the study design, population, interventions, and, if applicable, trial acronym	Line 1-4	
Trial registration	#2a	Trial identifier and registry name. If not yet registered, name of intended registry	Line 57	
Trial registration: data set	#2b	All items from the World Health Organization Trial Registration Data Set	Line 57	Information found at ClinicalTrial.gov and in the Swedish Study Protocol currently only available in Swedish, which is sufficient for the conduct of the clinical trial. However, we intent to translate the

For peer review only - http://bmjopen.bmj.com/site/about/guidelines.xhtml

				protocol to English but due to covid-19 the process is delayed.
Protocol version	#3	Date and version identifier		Not included in manuscript but in the Swedish Study Protocol
Funding	#4	Sources and types of financial, material, and other support	Line 405-409	
Roles and responsibilities: contributorship	#5a	Names, affiliations, and roles of protocol contributors	Line 393-403	More details are included in the Swedish Study Protocol approved by the Swedish Ethical Review Authority and the Swedish Medical Products Agency.
Roles and responsibilities: sponsor contact information	#5b	Name and contact information for the trial sponsor		Not included in manuscript but in the Swedish Study Protocol. Academic investigator-initiated trial (no pharmaceutical company involved).
Roles and responsibilities: sponsor and funder	#5c	Role of study sponsor and funders, if any, in study design; collection, management, analysis, and interpretation of data; writing of the report; and the decision to submit the report for publication, including whether they will have ultimate authority over any of these activities	Line 408-409	
Roles and responsibilities: committees	#5d	Composition, roles, and responsibilities of the coordinating centre, steering committee, endpoint adjudication committee, data management team, and other individuals or groups overseeing the trial, if applicable (see Item 21a for data monitoring committee)	Line 271-273	The study has no special committees as it is not required for this type of Phase II trial according to the Swedish Medical Products Agency.

For peer review only

**Introduction**			Line 75-119	
Background and rationale	#6a	Description of research question and justification for undertaking the trial, including summary of relevant studies (published and unpublished) examining benefits and harms for each intervention	Line 80-117 and line 326-331	
Background and rationale: choice of comparators	#6b	Explanation for choice of comparators	Line 291-297	Included in “Material and analysis – Data analysis plan”.
Objectives	#7	Specific objectives or hypotheses	Line 117-119	
Trial design	#8	Description of trial design including type of trial (eg, parallel group, crossover, factorial, single group), allocation ratio, and framework (eg, superiority, equivalence, non-inferiority, exploratory)	Line 122	To adhere to word limitation and avoid repetition this is declared once in the first lines of the section “Methods and analysis”.
**Methods: Participants, interventions, and outcomes**				
Study setting	#9	Description of study settings (eg, community clinic, academic hospital) and list of countries where data will be collected. Reference to where list of study sites can be obtained	Line 126-136	
Eligibility criteria	#10	Inclusion and exclusion criteria for participants. If applicable, eligibility criteria for study centres and individuals who will perform the interventions (eg, surgeons, psychotherapists)	Line 138-140, Table 1	

For peer review only

Interventions: description	#11a	Interventions for each group with sufficient detail to allow replication, including how and when they will be administered	Line 159-173, Table 2	More details are included in the Swedish Study Protocol.
Interventions: modifications	#11b	Criteria for discontinuing or modifying allocated interventions for a given trial participant (eg, drug dose change in response to harms, participant request, or improving / worsening disease)	Line 185-207	Not included in detail in the manuscript but in the Swedish Study Protocol.
Interventions: adherance	#11c	Strategies to improve adherence to intervention protocols, and any procedures for monitoring adherence (eg, drug tablet return; laboratory tests)	Figure 1	Not included in detail in the manuscript but in the Swedish Study Protocol.
Interventions: concomitant care	#11d	Relevant concomitant care and interventions that are permitted or prohibited during the trial	Figure 1, line 174-175 and line 185-192	Not included in detail in the manuscript but in the Swedish Study Protocol.
Outcomes	#12	Primary, secondary, and other outcomes, including the specific measurement variable (eg, systolic blood pressure), analysis metric (eg, change from baseline, final value, time to event), method of aggregation (eg, median, proportion), and time point for each outcome. Explanation of the clinical relevance of chosen efficacy and harm outcomes is strongly recommended	Line 52-53, 276-297	
Participant timeline	#13	Time schedule of enrolment, interventions (including any run-ins and washouts), assessments, and visits for participants. A schematic diagram is highly recommended (see Figure)	Figure 1	More detailed flowchart is available for the study team in the Swedish Study Protocol approved by the Swedish Ethical Review Authority and the Swedish Medical Product Agency.

For peer review only

Sample size	#14	Estimated number of participants needed to achieve study objectives and how it was determined, including clinical and statistical assumptions supporting any sample size calculations	Line 287-296	
Recruitment	#15	Strategies for achieving adequate participant enrolment to reach target sample size	Line 126-131 and line 294-296	
**Methods: Assignment of interventions (for controlled trials)**				
Allocation: sequence generation	#16a	Method of generating the allocation sequence (eg, computer-generated random numbers), and list of any factors for stratification. To reduce predictability of a random sequence, details of any planned restriction (eg, blocking) should be provided in a separate document that is unavailable to those who enrol participants or assign interventions	Line 291-302	
Allocation concealment mechanism	#16b	Mechanism of implementing the allocation sequence (eg, central telephone; sequentially numbered, opaque, sealed envelopes), describing any steps to conceal the sequence until interventions are assigned	Line 162-165 and line 297-302	
Allocation: implementation	#16c	Who will generate the allocation sequence, who will enrol participants, and who will assign participants to interventions	Line 162-165	Not included in detail in the manuscript but in the Swedish Study Protocol.

For peer review only

Blinding (masking)	#17a	Who will be blinded after assignment to interventions (eg, trial participants, care providers, outcome assessors, data analysts), and how	Line 122 and line 162-165	The study is an academic investigator-initiated open-label phase II trial with no blinding after assignment to interventions.
Blinding (masking): emergency unblinding	#17b	If blinded, circumstances under which unblinding is permissible, and procedure for revealing a participant's allocated intervention during the trial		Not applicable.
**Methods: Data collection, management, and analysis**				
Data collection plan	#18a	Plans for assessment and collection of outcome, baseline, and other trial data, including any related processes to promote data quality (eg, duplicate measurements, training of assessors) and a description of study instruments (eg, questionnaires, laboratory tests) along with their reliability and validity, if known. Reference to where data collection forms can be found, if not in the protocol	Figure 1, line 158-162, line 174-213, Table 3 and line 273-275.	Not included in detail in the manuscript but in the Swedish Study Protocol.
Data collection plan: retention	#18b	Plans to promote participant retention and complete follow-up, including list of any outcome data to be collected for participants who discontinue or deviate from intervention protocols	Line 208-213 and line 299-302.	Lost to follow-up for patients with TB in Sweden is extremely rare. In addition, we have access to the Swedish national tuberculosis registry that keeps track on long-term outcome of every active case. More details are included in the Swedish Study Protocol.
Data management	#19	Plans for data entry, coding, security, and storage, including any related processes to		Not included in detail in the manuscript but in the Swedish Study Protocol

For peer review only

		promote data quality (eg, double data entry; range checks for data values). Reference to where details of data management procedures can be found, if not in the protocol		approved by the Swedish Ethical Review Authority and the Swedish Medical Products Agency.
Statistics: outcomes	#20a	Statistical methods for analysing primary and secondary outcomes. Reference to where other details of the statistical analysis plan can be found, if not in the protocol	Line 277-297	More details included in the Study Protocol.
Statistics: additional analyses	#20b	Methods for any additional analyses (eg, subgroup and adjusted analyses)		Not applicable for this phase II trial.
Statistics: analysis population and missing data	#20c	Definition of analysis population relating to protocol non-adherence (eg, as randomised analysis), and any statistical methods to handle missing data (eg, multiple imputation)	Line 297-299, 305-306	Not included in detail in the manuscript but in the Swedish Study Protocol.
**Methods: Monitoring**				
Data monitoring: formal committee	#21a	Composition of data monitoring committee (DMC); summary of its role and reporting structure; statement of whether it is independent from the sponsor and competing interests; and reference to where further details about its charter can be found, if not in the protocol. Alternatively, an explanation of why a DMC is not needed	Line 271-273 and line 384-386	The support unit for clinical trials, Forum Östergötland, Linköping University is the independent study monitoring unit that will ensure adherence to the Study Protocol (including GCP and Declaration of Helsinki) throughout the study.
Data monitoring: interim analysis	#21b	Description of any interim analyses and stopping guidelines, including who will have access to these interim results and make the final decision to terminate the trial		Not applicable, interim analysis is not needed. More details are included in the Swedish Study Protocol regarding criteria for the sponsor to stop the study.

For peer review only

Harms	#22	Plans for collecting, assessing, reporting, and managing solicited and spontaneously reported adverse events and other unintended effects of trial interventions or trial conduct	Line 185-213 and line 388-389	
Auditing	#23	Frequency and procedures for auditing trial conduct, if any, and whether the process will be independent from investigators and the sponsor		When the Swedish Medical Product Agency approves an interventional clinical trial, audits may be performed by them at any time. Details included in the Swedish Study Protocol.
**Ethics and dissemination**				
Research ethics approval	#24	Plans for seeking research ethics committee / institutional review board (REC / IRB) approval	Line 54-55 and line 349-351	
Protocol amendments	#25	Plans for communicating important protocol modifications (eg, changes to eligibility criteria, outcomes, analyses) to relevant parties (eg, investigators, REC / IRBs, trial participants, trial registries, journals, regulators)	Line 388-389	
Consent or assent	#26a	Who will obtain informed consent or assent from potential trial participants or authorised surrogates, and how (see Item 32)	Line 358-359	
Consent or assent: ancillary studies	#26b	Additional consent provisions for collection and use of participant data and biological specimens in ancillary studies, if applicable		Not included in the manuscript but is described in patient study information/informed consent.
Confidentiality	#27	How personal information about potential and enrolled participants will be collected, shared,		Not included in the manuscript but is described in patient study information/informed consent.

For peer review only

		and maintained in order to protect confidentiality before, during, and after the trial		
Declaration of interests	#28	Financial and other competing interests for principal investigators for the overall trial and each study site	Line 411	
Data access	#29	Statement of who will have access to the final trial dataset, and disclosure of contractual agreements that limit such access for investigators		Not included in detail in the manuscript but in the Swedish Study Protocol.
Ancillary and post trial care	#30	Provisions, if any, for ancillary and post-trial care, and for compensation to those who suffer harm from trial participation		Not included in detail in the manuscript but in the Swedish Study Protocol and the patient study information/informed consent.
Dissemination policy: trial results	#31a	Plans for investigators and sponsor to communicate trial results to participants, healthcare professionals, the public, and other relevant groups (eg, via publication, reporting in results databases, or other data sharing arrangements), including any publication restrictions	Line 387-388 and line 309-310	
Dissemination policy: authorship	#31b	Authorship eligibility guidelines and any intended use of professional writers	Line 393-403	The section is written according to authorship eligibility guidelines.
Dissemination policy: reproducible research	#31c	Plans, if any, for granting public access to the full protocol, participant-level dataset, and statistical code	Line 391	The Study Protocol is currently only available in Swedish.
**Appendices**				

For peer review only

Informed consent materials	#32	Model consent form and other related documentation given to participants and authorised surrogates		Details not included in the manuscript but is available in the Study Protocol with the attached patient study information/consent form.
Biological specimens	#33	Plans for collection, laboratory evaluation, and storage of biological specimens for genetic or molecular analysis in the current trial and for future use in ancillary studies, if applicable	Line 216-265	

It is strongly recommended that this checklist be read in conjunction with the SPIRIT 2013 Explanation & Elaboration for important clarification on the items. Amendments to the protocol should be tracked and dated. The SPIRIT checklist is copyrighted by the SPIRIT Group under the Creative Commons "Attribution-NonCommercial-NoDerivs 3.0 Unported" license. This checklist can be completed online using https://www.goodreports.org/, a tool made by the EQUATOR Network in collaboration with Penelope.ai

For peer review only
